# Accumulation of Siglec10^+^CX3CR1^+^ Macrophages in the Tumor Microenvironment of Glioblastomas

**DOI:** 10.1002/eji.70180

**Published:** 2026-04-20

**Authors:** Nico Andreas, Lucas Gath, Paul Mike Jordan, Saskia Steiner, Oliver Werz, Nazife Dinc, Peter Baumgarten, Christian Senft, The OncoSurgeJena Team, Falko Schwarz, Nazeer Aboud, Ramazan Dalkilic, Sergio A. Calero Martinez, Katharina Klumbies, Aaron Lawson McLean

**Affiliations:** ^1^ Department of Neurosurgery Jena University Hospital Friedrich Schiller University Jena Jena Germany; ^2^ Comprehensive Cancer Center Central Germany Jena/Leipzig Germany; ^3^ Department of Pharmaceutical/Medicinal Chemistry Friedrich Schiller University Jena Institute of Pharmacy Jena Germany; ^4^ Jena Center for Soft Matter (JCSM) Friedrich Schiller University Jena Jena Germany

**Keywords:** cancer, immunopathology, innate immunity, macrophages, tumor immunology

## Abstract

Glioblastoma (GBM) is one of the most aggressive primary brain tumors in adults. Despite various strategies, including differential single‐cell gene expression analyses between GBM and healthy tissues, none of the resulting findings could benefit the development of promising therapies, yet. Various macrophage populations coevolve in the tumor microenvironment of GBMs and not only support tumor immune evasion but also tumor spreading throughout the surrounding tissue. To distillate unique immune cell features of GBMs, we compared their immune cell composition with other central nervous system (CNS) tumor entities, in particular meningiomas (MNGs), non‐GBM gliomas, and metastases (MTS). We could identify a macrophage population characterized by the coexpression of CX3CR1 and Siglec10, representing a potential indicator population of GBMs. This GBM‐specific macrophage subset might be supported by a glioma‐characteristic lipid mediator (LM) milieu of enriched docosahexaenoic acid (DHA) and its lipoxygenase (LOX)‐derived metabolites. Moreover, the GBM tumor microenvironment (TME) is enriched in arachidonic acid (AA)‐derived cyclooxygenase (COX) products prostaglandins (PG) E_2_ and F_2α_ in combination with enhanced CD24 expression. By our comparative approach, the data hint toward a pro‐tumorigenic Siglec10^+^CX3CR1^+^ macrophage population depending on the characteristic tumor microenvironment (TME) of highly malignant GBMs.

## Graphical abstract

By comparing various brain tumors, we identified a CX3CR1^+^ Siglec10^+^ double‐positive macrophage subpopulation specifically enriched in highly malignant Glioblastomas (GBMs), wherein they accumulated in metabolically active tumor areas. Thus, they potentially serve as a diagnostic indicator population for GBMs and as targets of future GBM‐specific treatment strategies.

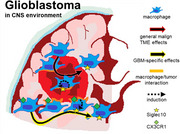

## 1 Introduction

Glioblastoma (GBM) is one of the most aggressive brain tumors in adults, with a median survival of approximately 15 months despite multimodal therapy [1]. Compared with other CNS tumor entities, the incidence of GBMs increases with age [2]. While many characteristics of GBMs have been attributed to therapy resistance, for example, tumor cell interaction with the local microenvironment, tumor cell migration and invasiveness, as well as metabolic alterations [3], none of them have led to the development of improved GBM therapies, yet [4]. Even therapies effective in treating non‐CNS tumors showed no effect on GBMs, which suggests that GBMs possess an exclusive resistance mechanism [5]. One major obstacle is the wide variety of cell types identified within the GBM environment, which led to the outdated name attribute “multiformis” in the past. Depending on the GBM region of the analyzed sample, not only can oligodendrocytes, astrocytes or astroglia be enriched (close to tumor core) or do neuronal cells predominate (tumor infiltration zone), but also the GBM‐associated canonical signatures vary from “classical” or “proneural” signatures (core regions) to “neural” signatures (infiltration zone) [6]. The active part of the tumor core defined by high protoporphyrine‐IX (PPIX) signals, a metabolite of 5‐aminolevulinic acid (5‐ALA) frequently used to assist neurosurgical resection via accumulated fluorescence in GBM tumor cells [7], even matches the “mesenchymal” subtype, whereas necrotic and PPIX‐negative part of the tumor core matches mostly the “proneural” or “neural” classifiers [6]. To take these multiform GBM characteristics to the extreme, various brain macrophage populations have been documented interacting with these variations of GBM tumor cells [8].

Brain macrophages comprise distinct resident brain cell populations, including microglia, border‐associated macrophages (BAM), or monocyte‐derived macrophages (MDMs), exerting differential functions related to their origins [9, 10]. While microglia arise from embryonal yolk sac progenitors migrating to the brain, BAM progenitors are derived from yolk sac and fetal liver and migrate to different anatomical sites [10, 11]. However, BAMs are maintained not only by long‐lived residual precursors or by blood‐borne monocytes, but also by monocytes from the adjacent skull or vertebral bone marrow [10, 11]. Microglia and BAMs control brain development and homeostasis, whereas blood monocyte‐derived macrophages accumulate at CNS sites upon pathological environmental changes, that is, during glioma development [12, 13, 14]. While in tumor‐free brain tissue, microglia are predominant, within the tumor microenvironment (TME), tumor‐associated macrophages (TAMs) of non‐microglial origin represent the most abundant stromal cell type [14, 15] and are thought to drive glioma development and progression [12, 16]. TAMs are recruited in early phases of the GBM genesis and are located mainly in the perivascular regions [17]. They potentially hinder immune checkpoint therapies and support tumor growth, survival, and angiogenesis [18], and matrix remodeling [15]. It is highly likely that depending on the tumor entity, TAMs likely differ in surface characteristics and function depending on the tumor entity. However, such assumptions might not yet have been adequately addressed due to the lack of surface markers for identification [9]. However, TAMs coevolving with tumors in the TME support the evasion from conventional treatments or immunotherapy [19]. Approaches to modulate TAMs as a therapeutic strategy have included the targeting of TAM differentiation factors or TAM functional molecules, and even employing genetically engineered TAMs [20]. However, the challenge in GBM treatment is still to maintain a pro‐inflammatory setting over a protumorigenic setting, to keep therapeutic approaches effective [20].

Apart from immunotherapeutic strategies, targeting the lipid environment in GBMs is a promising potential concept. The GBM TME exhibits an altered lipid metabolism and thereby adapts to its neoplastic environment of limited nutrients and limits pro‐inflammatory reactions [21, 22]. Depending on the substrate availability, a locally increased formation of particular oxidized PUFAs is mediated by enzymes like 15‐lipoxygenase (15‐LOX) [23] or 5‐LOX [24]. The most abundant fatty acid in the human brain is docosahexaenoic acid (DHA), and therefore, the formation of bioactive lipid mediators from DHA seems to be predominant by default [25]. The 15‐LOX‐dependent DHA metabolite 17‐hydroxydocosahexaenoic acid (17‐HDHA) is a pro‐resolving mediator that enhances tissue‐remodeling macrophage responses [26] and reduces inflammatory cytokine responses in adipose tissues [27]. Among the 5‐LOX products, 7‐HDHA derived from DHA is specifically increased upon tissue‐remodeling macrophage activation [28]. It is a high‐affinity ligand of PPARα and stimulates neuronal growth and branching, and supports neuronal plasticity [29]. Thus, a regulatory circle is established that mediates neoplastic brain tissue, local tissue remodeling, and immune suppression against the background of programmed cell death.

Here, we analyzed the immune cell composition in different parts of GBMs, non‐GBM gliomas, meningiomas (MNG), and metastases (MTS), and identified a macrophage population specifically enriched in GBMs. This macrophage population is characterized by the coexpression of CX3CR1 and Siglec10 and might be specifically supported by a glioma‐characteristic lipid mediator (LM) milieu of enriched DHA, the 15‐LOX product 17‐HDHA, and the COX products PGE_2_ and PGF_2a_ in combination with CD24 expression. By our comparative approach, our data hint toward a pro‐tumorigenic macrophage population depending on the characteristic TME of highly malignant GBMs.

## 2 Material and Methods

### 2.1 Tissue Sampling and Fluorescence Guidance

We prospectively obtained tissue samples of patients undergoing craniotomy for brain tumors, and included patients with suspected gliomas, MTS, as well as MNG. As this is a prospective/exploratory study, we did not apply any inclusion or exclusion criteria to any of the patients’ samples. In line with that, we did not consider sex or age as a variable for our analysis. Detailed information on distribution by sex and age among our patient cohort is shown in Table S1. No blinding or randomization methods were applied. Glioma and MTS (glioma‐suspected diagnosis) patients were orally supplied with Gliolan (5‐amino‐4‐oxopentanoic acid hydrochloride = 5‐ALA) at 20 mg/kg body weight approximately 4 h before surgery. Malignant tumor cell metabolism leads to the accumulation of Protoporphyrin‐IX (PPIX) within the tumor cells. During surgery, tumor cell fluorescence was induced for the detection of intratumoral heterogeneity using a surgical microscope (Kinevo 900, Zeiss, Oberkochen/Germany) with special filters as described previously [7].

### 2.2 Buffers and Medium

Phosphate‐buffered saline (PBS) was prepared in H_2_O by addition of 8 g/L NaCl, 0.2 g/L KCl, 1.42 g/L Na_2_HPO_4_, and 0.24 g/L KH_2_PO_4_. Erythrocytes were lysed with ery lysis buffer containing 8.3 g NH_4_Cl, 1 g KHCO_3_, 18.6 mg EDTA, in H_2_O, pH 7.2–7.4. PBA/E, a buffer composed of PBS, bovine fetal serum, and azide (sodium salt) with the addition of EDTA (PBS, 2% FBS, 0.1% NaN3, 2 mM EDTA), was used to prepare the single cell solution for flowcytometric analysis. Fully supplemented DMEM (fsDMEM) was composed of DMEM (1×) high glucose + L‐glutamine—sodium pyruvate (Thermo Fisher Scientific Inc.) containing 10% fetal bovine serum (FBS, Thermo Fisher Scientific Inc.) and 10,000 U/mL penicillin‐streptomycin (Thermo Fisher Scientific Inc.).

### 2.3 Digestion of Tumor Samples

100 mg tissue samples were minced per 1 mL tissue digestion mix: PBS containing 1 mg/mL hyaluronidase (Sigma‐Aldrich Chemie GmbH), 1 mg/mL collagenase I (Worthington Biochemical Corporation), 1 mg/mL collagenase IV (Worthington Biochemical Corporation), and 0.2 mg/mL DNase I (Roche Holding AG). The dissociation mix was shaken at 37°C for 1 h. Subsequently, a single cell suspension was generated by passing the digested tissue through a 70 µm cell strainer (Thermo Fisher Scientific Inc.). Erythrocytes were lysed with ery lysis buffer at RT for 5 min. Cells were counted using a Neubauer counting chamber.

### 2.4 Primary Tumor Cell Cultures

For the primary cell culture, cells were plated at a concentration of 300,000/cm^2^ in cell culture containments. When the cultures reached 90% confluency, cells were detached with 0.05% (v/v) Trypsin/EDTA solution (Thermo Fisher Scientific Inc.) at 37°C for 5 min. Digestion was stopped with fsDMEM, and cells were plated in a new cell culture containment at 15,000 cells/cm^2^ to further expand the cells. Cells were used for experiments at passage (P) 4 until P10.

### 2.5 Preparation of Peripheral Blood Mononuclear Cells and Monocytes

Leukopacks (buffy coats) were diluted with PBS at 1:5 and subsequently layered on a Bicoll matrix (1.077 g/mL, Bio&SELL GmbH). Upon centrifugation at 800×*g* at RT, the intermediate layer containing peripheral blood mononuclear cells (PBMCs) was isolated and washed in fsDMEM. Cell count was determined with a Neubauer counting chamber. To isolate monocytes from PBMCs, 10^8^ PBMCs were labelled with anti‐human CD14 antibodies coupled to magnetic beads (Miltenyi Biotec). Subsequently, CD14^+^ monocytes were purified via LS column magnetic fields (Miltenyi Biotec). Purified monocytes were washed twice with fsDMEM, and the cell count was determined. If indicated, PBMCs were cultured at 2 × 10^6^ cells/well in a 24‐well plate in 500 µL RPMI and stimulated with 10 µM PGD2, PGE2, or PGF2A or with a representative volume of EtOH as solvent control for 24 h. Subsequently, cells were recovered and analyzed for CD45, CD14, and Siglec10 expression by flow cytometry.

### 2.6 Co‐Culture of Tumor Cells and PBMC/Monocytes

Before co‐culture, 50,000 tumor cells/well were seeded into 24‐well plates and cultured for 24 h. Subsequently, either 10^6^ PBMCs or 3 × 10^5^ monocytes/macrophages were added to the cultures as indicated. After 96 h, supernatants containing floating cells were preserved, while adherent cells were detached with tissue digestion mix. For flow cytometric analysis, the preserved supernatant was pooled with the associated detached cells.

### 2.7 Flowcytometry

2 × 10^6^ cells were resuspended in 25 µL mouse serum and incubated at 4°C for 5 min. Subsequently, antibodies for flow cytometry were added according to manufacturers’ recommendations, and all preparations were incubated in the dark at 4°C for 20 min. Staining was stopped by the addition of 1 mL of PBA/E buffer and washed twice before analysis using centrifugation steps of 200×*g*/4°C/10 min. To stain the dead cells, all pellets were reconstituted in 400 mL PBA/E containing 1 µg/mL DAPI, followed by analysis on a LSR Fortessa (BD Bioscience). A stable performance was controlled by frequent CS&T checks as recommended by BD. The samples were acquired by keeping PMT voltages and compensations constant to guarantee data comparability. The following antibodies from Miltenyi Biotech were used as recommended by the supplier: anti‐human CD31‐APC/Vio770 (clone REA730, RRID: AB_2657288), anti‐human CD326‐Vio Bright 720 (clone REA764, RRID: AB_2657498). Following antibodies from Biolegend were used as recommended by the supplier: anti‐human CD3‐BV510 (clone SK7, RRID: AB_2563703), anti‐human CD45‐BV650 (clone: HI30, RRID: AB_2562498), anti‐human CD19‐PerCP/Cy5.5 (clone: HIB19, RRID: AB_2275547), anti‐human CD66b‐APC (clone: G10F5, RRID: AB_2566606), anti‐human CD14‐AF700 (clone: M5E2, RRID: AB_493747), anti‐human CD68‐BV785 (clone: Y1/82A, RRID: AB_2800879), anti‐human CD56‐PE/Cy5 (clone: 5.1H11, RRID: AB_2564088), anti‐human γδTCR‐PE (clone: B1, RRID: AB_1089219), anti‐human TCRβ‐BV605 (clone: IP26, RRID: AB_2650818), anti‐human CD8‐APC/Cy7 (clone: SK1, RRID: AB_2044005), anti‐human CD4‐Spark PLUS UV395 (clone: SK3, RRID: AB_3097546), anti‐human CD45‐PE/Cy5 (clone: HI30, RRID: AB_314397), anti‐human CD24‐BV605 (clone: ML5, RRID: AB_2562287), anti‐human CD44‐BV785 (clone: BJ18, RRID: AB_2860873), anti‐human CD324‐PE/Cy7 (clone: 67A4, RRID: AB_2563095), anti‐human CD325‐PE (clone: 8C11, RRID: AB_10660824), anti‐human Siglec10‐PE/Cy7 (clone: 5G6, RRID: AB_2814275), anti‐human CD14‐PerCP/Cy5.5 (clone: M5E2, RRID: AB_893253), anti‐human Siglec10‐PE (clone: 5G6, RRID: AB_2270417), and anti‐human CX3CR1‐PE/Dazzle 594 (clone: 2A9‐1, RRID: AB_2687151). The following antibodies from ThermoFisher Scientific were used as recommended by the supplier: anti‐human CD45‐PE/Cy5.5 (clone: HI30, RRID: AB_10375162). For evaluation of the data, FlowJo 10.8.1 (BD) was used. A gating strategy was set up on PBMCs from healthy donors, as shown in Figure S1. These gates were then applied to all tumor samples for subsequent analyses.

### 2.8 Fluorescence Microscopy

Parts of the patient‐derived tumor samples were cryoconserved within 1 h after resection. To cut 20 µm slices, all samples were transferred into deionized water on Superfrost Plus Adhesion Microscope slides (Epredia Netherlands B.V., the Netherlands). Subsequently, slices were flushed with PBS and fixed with ice‐cold Aceton for 10 min. Upon washing, all samples were permeabilized with PBS‐T (0.25% Triton‐X‐100 in PBS) for 10 min. Upon repeated washing, all slices were blocked with 10% donkey serum in PBS for 1 h. Staining of respective antigens by primary antibodies in 10% donkey serum in PBS was done at 4°C overnight: mouse anti‐human CD68 (BioLegend, clone Y1/82A, 1:50), rabbit anti‐human Siglec10 (Bioss, polyclonal, 1:100) and rat anti‐human CX3CR1 (BioLegend, clone 2A9‐1, 1:10). Upon washing with PBS, AffiniPure Donkey Anti‐Rat IgG (H+L) Alexa Fluor 488 (Jackson ImmunoResearch, 1:400), goat anti‐Rabbit IgG (H+L) Cross‐Adsorbed Secondary Antibody, Alexa Fluor 568 (ThermoFisher Scientific, 1:1000), and Donkey anti‐Mouse IgG, Cy5 conjugated (MilliporeSigma, 1:400) was incubated on the slides at RT for another 2 h. Upon subsequent washing with PBS, nuclei were stained with Hoechst33258 dye (Merck, 1:1000) at RT for 5 min. After washing with PBS, all sections were covered with mounting medium and glass slides for microscopic analysis. All histological pictures were taken on an Axio Observer 7 (Zeiss, Germany).

### 2.9 LM Metabololipidomics

20–50 mg of each tumor entity in 20 µL/mg PBS was extracted using a FastPrep‐24 5G Lysis System (MP Biomedicals, Eschwege, Germany). After homogenization, lysates were mixed with the same volume of ice‐cold methanol. Samples were vortexed, and after centrifugation (5 min, 15,000 rpm, 4°C), the supernatant was filled up with methanol to a final volume of 3 mL. 10 µL of a deuterium‐labeled standard mix (containing 200 nM d8‐5‐HETE, d4‐LTB_4_, d5‐LXA_4_, d5‐RvD2, d4‐PGE_2_, and 10 µM d8‐AA [Cayman Chemicals]) was added to each sample. Proteins were precipitated, centrifuged, and to the supernatant, acidified H_2_O was added (1/9, v/v, final pH = 3.5). Samples were subjected to solid‐phase extraction using Sep‐Pak Vac 6cc 500 mg/6 mL C18 (Waters). After washing with H_2_O and n‐hexane, LM were eluted with methyl formiate. Samples were dried using an evaporation system (TurboVap LV) and resuspended in methanol‐water (50/50, v/v) for UPLC‐MS‐MS automated injections. LM profiling was analyzed with an Acquity UPLC system (Waters) and a QTRAP 5500 mass spectrometer (ABSciex) equipped with a TurboV Source and electrospray ionization. LM was eluted using an ACQUITY UPLC BEH C18 column (1.7 µm, 2.1 × 100 mm; Waters) at 50°C with a flow rate of 0.3 mL/min and a mobile phase consisting of methanol–water–acetic acid of 42:58:0.01 (v/v/v) that was ramped to 86:14:0.01 (v/v/v) over 12.5 min and then to 98:2:0.01 (v/v/v) for 3 min. The QTrap 5500 was operated in negative ionization mode using scheduled multiple reaction monitoring (MRM) coupled with information‐dependent acquisition. The scheduled MRM window was 60 s, optimized LM parameters were adopted, and the curtain gas pressure was set to 35 psi. The retention time and at least six diagnostic ions for each LM were confirmed by an external standard (Cayman). Quantification was achieved by calibration curves for each LM. Linear calibration curves were obtained for each LM and gave *r* ^2^ values of 0.998 or higher (for fatty acids, 0.95 or higher). Additionally, the limit of detection for each targeted LM was determined, as reported before [30]. The identity of low‐abundant analytes was confirmed by fragmentation‐pattern matching by re‐analysis on a QTrap 7500 mass spectrometer (Sciex, Framingham, MA, USA) controlled by SCIEX‐OS, and comparing the enhanced product ion scans of the biological sample with those of authentic standards. Arbitrary units were calculated sample‐wise as the relative amount of each separate LM to the average amount of all analyzed LMs.

### 2.10 Statistical Analysis

Sigmaplot 14 (SYSTAT Software GmbH, Germany) was used to perform statistical analysis. The Shapiro–Wilk test was used to test for normal distribution, and the Brown–Forsythe test was used to test for equal variance.

For comparison of sample groups from independent samples, the following tests were applied: If data were distributed normally, a two‐sided Student's *t*‐test was used for data with equal variance, or when the equal variance test failed, a Welch's *t*‐test was used. If the test for normal distribution failed, a Mann–Whitney *U*‐test was performed.

For comparing multiple groups from interdependent samples, one‐sided analysis of variance (ANOVA) was applied. If the grouped data were normally distributed and of equal variance, the Holm–Sidak's multiple comparison procedure (Holm–Sidak method) was performed to identify groups that were significantly different. If the data were not normally distributed or of unequal variance, the Kruskal–Wallis ANOVA on Ranks was performed. To reveal significantly different groups, the following tests were used: For data groups of equal sizes, the Student–Newman–Keuls multiple comparison was used, while for data groups of unequal sizes, the Dunn's multiple comparison procedure (Dunn's Method) was applied.

For identification of outliers, Grubb's outlier test was performed (*α* = 0.05).

All data are expressed as means with SEM unless otherwise indicated.

Significance is indicated as a *p‐*value for each probability of *p* = 0.05 or higher and less than *p* = 0.10. Otherwise, significance is either not indicated or by stars as follows: *p < 0.05, **p < 0.01, or ***p < 0.001.

## 3 Results

### 3.1 Accumulation of Myeloid Cells in PPIX‐Positive GBM Areas

**FIGURE 1:**
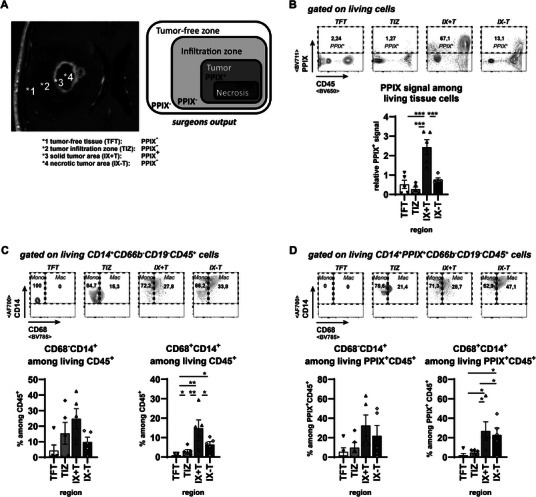
Monocytes and macrophages accumulate in tissues influenced by the GBM. Samples were acquired by visual guidance of the surgeon, assisted by PPIX fluorescence. (A) Indication of the localization of GBM samples on T2‐signal MRI pictures and association of reported PPIX signals: *1: PPIX‐negative Tumor‐free tissue (TFT); *2: PPIX‐negative Tumor infiltration zone (TIZ); *3: PPIX‐positive tumor bulk tissue (XI+T); *4: PPIX‐negative Tumor necrosis area (IX‐T). (B) Evaluation of the intraoperative PPIX‐guided tissue sampling by flow cytometry. (C, D) Analysis of CD68^−^CD14^+^ macrophages (left) and CD68^+^CD14^+^ monocytes (right) among living CD45^+^ cells (C) or PPIX^+^CD45^+^ cells (D) by flowcytometry. All data were collected from five different patients, and statistical analyses were done as described in the material and methods: *p < 0.05, **p < 0.01, ***p < 0.001.

Glioblastomas are primary tumors of heterogeneous cellular composition. Due to resulting spatial variations, sampling these tumors for analysis potentially yields varying cell compositions. To circumvent these variations and reproducibly target specific core structures of each analyzed GBM tissue, we utilized the fluorescence‐induced protoporphyrine‐IX (PPIX) signal combined with the surgeon's visual orientation. As shown in Figure 1A, we defined different zones of the GBM for analysis. We obtained samples from the PPIX‐negative tumor‐free entrance tissue (TFT, *1), from the PPIX‐negative diffuse tumor infiltration zone (TIZ, *2), from the PPIX‐positive tumor core (IX+T, *3), and from the central PPIX‐negative Tumor necrosis zone (IX‐T, *4). For verification, we analyzed the PPIX signal of each particular region by flow cytometry (FACS), and thereby, we could confirm the proper intrasurgical selection of IX+T and IX‐T tissues. Only tissue samples selected as PPIX^+^ contained cells with enhanced PPIX fluorescence signals (Figure 1B).

To investigate hematopoietic cell compositions in the GBM environment, we gated on cells expressing CD45, which is widely accepted as the major marker for HSC‐derived cells (Figure S2A). We could not detect any specific accumulation of CD45^+^ hematopoietic cells in one of the selected areas of the tumor environment (Figure S2B). However, to avoid the impact of a background induced by neuronal debris on the cellular frequencies, we restricted all further analysis to frequencies among CD45^+^ hematopoietic cells.

Among CD45^+^ hematopoietic cells, we identified separate immune cell populations via their major surface antigens. While CD19^+^ B lymphocytes did not preferentially accumulate in any tumor region, we could detect increased frequencies of CD66b^+^ granulocytes in the IX‐T area of the GBM tumor (Figure S3A). CD14^+^ myeloid cells were virtually absent in TFT areas but accumulated in areas closer to the tumor core, being most prominent in IX+T areas (Figure S3B). We could detect only minor frequencies of CD56^+^CD3^−^ NK cells or CD56^+^CD3^+^ NKT cells, which did not show any preferential accumulation at any distance from the tumor core (Figure S3C).

Comparable to NK and NKT cells, αβT and γδT cells did not preferentially accumulate in any of the GBM tumor areas (Figure S4A–C). In contrast to Th cells, a slight tropism to the tumor environment was observed for the Tc cell compartments (Figure S4E). To get a more detailed view of the most dominant immune cell subset among the central tumor parenchyma, CD14^+^ myeloid cells, we further discriminated between infiltrated monocytes and activated tumor‐associated microglia/macrophages (TAM) by CD68 expression levels. Of note, the strength of CD68 expression negatively correlates with prognostic survival of GBM patients [31]. Following the spatial distribution of general CD14^+^ cells, CD68^+^CD14^+^ TAMs and CD68^−^CD14^+^ monocytes were hardly detectable in TFT areas but started to accumulate in the tumor‐infiltration zone and ultimately represented the majority of hematopoietic cells within the core tumor regions (Figure 1C). Confirming a recent report [32], monocytes and macrophages constituted the vast majority of the PPIX^+^ CD45^+^ hematopoietic cells within the visually defined IX+T areas, but also within the visually defined IX‐T areas (Figure 1D), wherein only rare PPIX signals were detectable in FACS (Figure 1B).

### 3.2 GBM Maintain Enhanced CD24 Expression throughout Culture

Various reports demonstrate that microglia decrease their phagocytic potential when activated in the presence of glioma cells [33, 34, 35]. In the tumor microenvironment, “don't eat me” signals predominantly suppress the innate immune system [36]. Expression analysis using TCGA datasets via GEPIA [37] showed that among known signals of that family, high levels of transcripts for the CD24‐Siglec10 and the LILRB1‐HLA axis are associated with GBMs (Figure S5A). HLA genes have already been described to be very heterogeneous between GBM patients [15]. We instead chose the signaling axis with the highest upregulation in GBM compared with healthy control tissue: CD24‐Siglec10 (Figure S5A,B).

**FIGURE 2:**
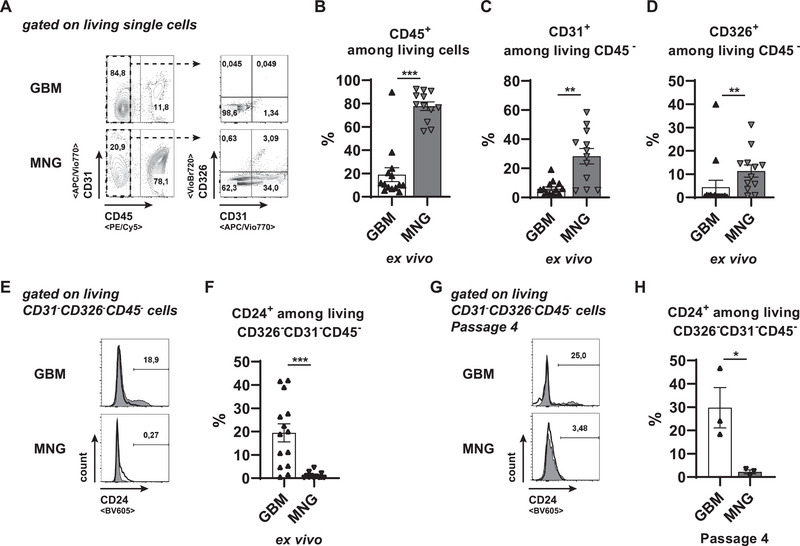
Enhanced CD24 expression remains stable among tumor cells from GBM. Tumor samples of the indicated entities were digested, and single cells were stained with antibodies against CD45, CD31, CD326, and CD24. (A) Representative gating strategy is shown. CD45 signals identify hematopoietic cells, and CD45^−^ cells were analyzed for expression of CD31 and CD326. (B–D) Summarized ex vivo analysis of CD45^+^ hemtopoietic cell frequencies (B) and of CD31^+^ (C) or CD326^+^ (D) cell frequencies among CD45‐ non‐hematopoietic cells. (E, F) Representative signals of CD24 among living CD31^−^CD326^−^CD45^−^ cells of the indicated tumor entities (E) with summarized data depicted in the diagram (F). (G, H) Obtained single cells were cultured to generate primary tumor cell cultures. Representative histogram for CD24 expression at passage 4 is shown among living CD31^−^CD326^−^CD45^−^ cells (G), and a summary of all cultures of the indicated entity is shown in the diagram (H). Statistics: ^**^ *p* < 0.01, ^***^ *p <* 0.001.

A problematic deception in the comparison of tumor cells with tumor‐free tissue cells is that tumor cells modify their surroundings, creating a unique neoplastic microenvironment by inducing spatial tissue stress or depletion of local nutrients, all of that being absent in healthy tumor‐free tissue. To investigate characteristics of GBM cells specific to their inherent malignant character, we compared the surface marker expression in the GBM environment to that of the neoplastic microenvironment of mostly “benign” meningiomas (Figure 2). To investigate the expression of CD24 in primary CNS tumors, we digested primary tumor tissues ex vivo and excluded hematopoietic cells via their CD45 signals, which were of significantly higher frequency in MNGs when compared with GBMs (Figure 2A,B). Furthermore, we could also detect more endothelial cells via CD31 signals (Figure 2A,C) and more epithelial cells by CD326 signals (Figure 2A,D) in MNGs compared with GBMs, which we excluded for subsequent analyses as well. Among the remaining CD31^−^CD326^−^CD45^−^ cells, the frequencies of CD24‐expressing cells were significantly enhanced in GBMs compared with MNGs (Figure 2E,F). When we compared other surface markers associated with GBM malignancy, namely CD44, CD324, and CD325, we did not detect any difference in surface marker expression between GBM and MNG tumor cells (Figure S6). While the expression of CD325 was maintained upon culture of the tumor cells over several passages (Figure S6K,L), CD44 (Figure S6C,D), and CD324 (Figure S6G,H) were even upregulated *in vitro*. Of note, the expression of CD24 among cultured GBM tumor cells was maintained throughout passage 4, while its expression remained low on cultured MNG tumor cells (Figure 2G,H). Collectively, GBM cells do maintain their CD24 expression during cell culture despite an increase in other tumor markers.

### 3.3 GBM Tumor Cells Enhance Siglec10 Expression in Myeloid Cells

**FIGURE 3:**
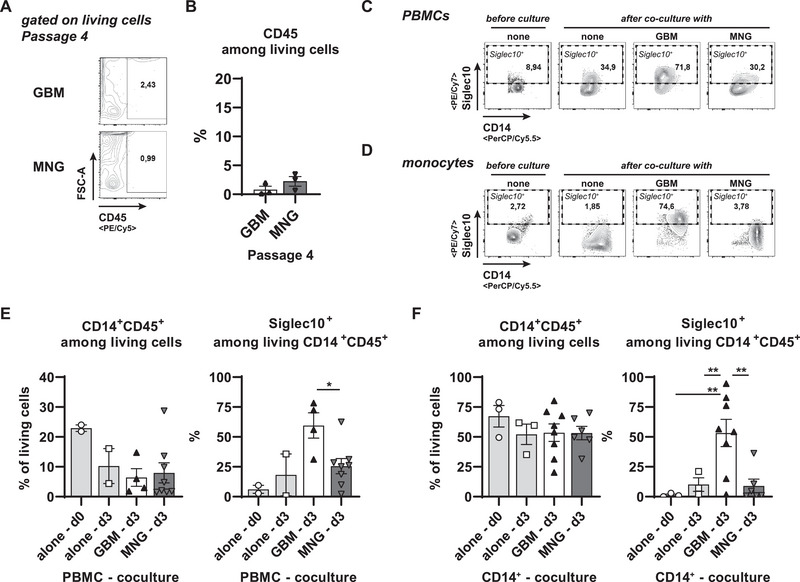
GBM tumor cells possess an intrinsic capacity to induce Siglec10+ monocytes. (A, B) Single cells from GBMs or MNGs were stained for CD45. Representative FACS plots (A) and summarized data from all analyses are shown (B, *n* = 3 independent biological samples). (C, E) PBMCs were isolated from healthy donors and cocultured with 50,000 primary tumor cells beyond passage 4 at a 20:1 ratio. After 3 days, cells were detached and analyzed for signals for CD45, CD14, and Siglec10 expression by flow cytometry. Representative FACS plots are shown in (C), and the diagrams summarize the results of PBMCs (E) from two different donors co‐cultured with cells from MNG samples (4 separate donors) or from GBM samples (2 separate donors). (D, F) CD14+ monocytes were isolated from healthy PBMCs and cocultured with 50,000 primary tumor cells beyond passage 4 at a 6:1 ratio. After 3 days, all cultured cells were detached and analyzed for signals for CD45, CD14, and Siglec10 expression by flow cytometry. Representative FACS plots are shown in (D), and the diagrams summarize the results of CD14^+^ PBMCs (F) from three different donors co‐cultured with cells from MNG samples (three separate donors) or from GBM samples (four separate donors). Statistics: ^*^ *p* < 0.05, ^**^ *p* < 0.01.

To analyze the capacity of GBM tumor cells to regulate Siglec10 expression on monocytes/macrophages, we analyzed CD14^+^CD45^+^ myeloid cells in direct co‐culture with tumor cells. Because CD45^+^ hematopoietic cells were virtually absent in tumor cell cultures at passage P4 or later (Figure 3A,B), we purified PBMCs (Figure 3C,E) or CD14^+^ monocytes (Figure 3D,F) from allogenic healthy blood donors and co‐cultured them with primary tumor cells from cultures between passage P4 and P10.

Upon co‐culture of PBMCs with tumor cells, we did not observe any overrepresentation of CD45^+^ remaining in the cultures as GBM and MNG cultures (Figure S7A,B). When we analyzed the subset distribution among the remaining CD45^+^ culture cells in tumor cell co‐cultures, we could only detect a slight decrease in NK cell frequencies compared with PBMCs cultured alone (Figure S7D). The frequencies of all other analyzed PBMC subsets remained comparable with or without tumor cells (Figure S7), including CD14^+^ myeloid cells (Figure 3C,E). The ligand for CD24 is Siglec10, which is expressed by CD45^+^ hematopoietic cells [38]. When we compared the frequency of Siglec10^+^ cells among CD14^+^ subsets of the co‐cultured PBMCs, we could detect an upregulation of Siglec10 among cells co‐cultured with GBM tumor cells, but neither in cells co‐cultured with MNG tumor cells nor among PBMCs cultured alone (Figure 3E). To exclude secondary effects induced by adaptive immune cells within the co‐cultured PBMCs, we enriched PBMCs for CD14^+^ cells by MACS and co‐cultured them with tumor cells from GBMs or MNGs. Confirming previous findings, no altered maintenance of myeloid cells could be observed between all conditions tested (Figure 3F). When we analyzed the levels of Siglec10 among co‐cultured purified CD14^+^ myeloid cells, we detected a strong increase in Siglec10‐expressing myeloid cells in conditions with GBM tumor cells, but not with MNG tumor cells (Figure 3E). Collectively, GBM tumor cells contain the capacity to induce Siglec10‐expressing myeloid cells even when they have been cultured before for more than three passages *in vitro*. Of note, this correlates with the CD24^+^ frequencies among GBM tumor cells, which, in contrast to CD44 or CD324, remained stable (Figure 2; Figure S6).

To test whether the accumulation of Siglec10^+^CD14^+^ myeloid cells in the presence of GBM tumor cells *in vitro* represents their status *in vivo*, we analyzed various CNS tumor entities ex vivo directly after resection. Macrophages migrate throughout tissues and can acquire tissue‐specific plasticity. Therefore, we not only compared MNG and GBMs, but also included non‐GBM gliomas (“Glioma”) and brain metastasis of diverse entities in our analysis. An overview of all entities is shown in Table S1. Confirming earlier observations (Figure 2B), we found strongly enriched CD45^+^ hematopoietic cells among living cellular structures of MNGs, but not among other tumor entities (Figure S8A,B). Reflecting our *i* *n* *vitro* findings, higher Siglec10^+^ frequencies were detected among hematopoietic CD45^+^ GBM cells compared with CD45^+^ MNG cells (Figure S8C,D). Notably, Siglec10^+^ frequencies among CD45^+^ cells in brain metastases and non‐GBM glioma ranged between those of GBMs and MNGs (Figure S8C,D).

### 3.4 GBMs Accumulate Enhanced Levels of Siglec10‐Expressing Macrophages

**FIGURE 4:**
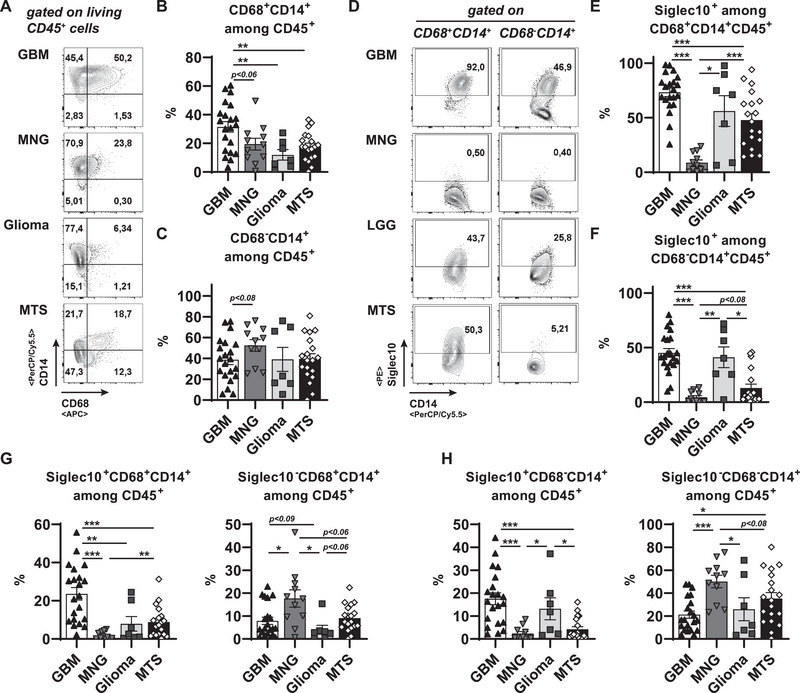
Preferential accumulation of Siglec10^+^ macrophages in GBM tumor environment. Tumor samples of the indicated entities for (GBM: *n* = 21; MNG: *n* = 12; Glioma: *n* = 7; MTS: *n* = 20) were digested, and single cells were stained with antibodies against CD45, CD14, CD68, and Siglec10. (A) Representative dot plots were pregated on living CD45 hematopoietic cells and subsequently plotted for CD14 and CD68. (B, C) CD68^+^CD14^+^ (B) and CD68^−^CD14^+^ (C) cell frequencies were summarized among living CD45^+^ hematopoietic cells. (D) Representative plots for Siglec10 frequencies among living CD68^+^CD14^+^ (left panels) or CD68^−^CD14^+^ (right panels) CD45^+^ subsets of the indicated tumor entities. (E, F) Summarized Siglec10 frequencies among CD68^+^CD14^+^ (E) or CD68^−^CD14^+^ (F) CD45^+^ subsets of the indicated entities. (G) Siglec10^+^ (left) or Siglec10^−^ (right) CD68^+^CD14^+^ cell frequencies among all living CD45^+^ hematopoietic cells were summarized for the indicated entities. (H) Siglec10^+^ (left) or Siglec10^−^ (right) CD68^−^CD14^+^ cell frequencies among all living CD45^+^ hematopoietic cells were summarized for the indicated entities. Statistics: ^*^ *p* < 0.05, ^**^ *p* < 0.01, ^***^ *p* < 0.001.

To ensure a more defined comparison, we continued our analysis on myeloid subsets among living CD45^+^ cells of the investigated CNS tumor entities (Figure 4A). GBMs accumulated more CD68^+^CD14^+^ TAMs than gliomas and MTS, but showed just a trend to accumulate more than MNGs (Figure 4B). Of note, GBMs, non‐GBM‐gliomas, and MTS did not differ in frequencies of CD68^−^CD14^+^ myeloid cells (Figure 4C). In line with the observed accumulation of Siglec10^+^ myeloid cells in primary GBM cell culture *i* *n* *vitro* (Figure 3E,F), we detected strongly enhanced frequencies of Siglec10‐expressing CD68^+^CD14^+^ and CD68^−^CD14^+^ CD45^+^ cells in GBM samples when compared with MNGs (Figure 4D–F). While we did not detect any differences in Siglec10^+^ frequencies between myeloid populations from GBMs and non‐GBM‐gliomas, the frequencies of MTS‐derived Siglec10^+^ myeloid cells were less than that of GBMs, but more than that of MNGs (Figure 4D–F). When we calculated the frequency of Siglec10^+^ cells related to their superior myeloid population, the GBM had significantly more Siglec10^+^CD68^+^CD14^+^ TAMs than any of the other entities (Figure 4G). Conversely, Siglec10^−^CD68^+^CD14^+^ were highest among MNGs CD45^+^ hematopoietic cells (Figure 4G). All gliomas accumulated much more Siglec10^+^CD68^−^CD14^+^ monocytes than MTSs and MNGs, which had only very low levels (Figure 4H). Of note, all observed differences did not emerge at the expense of the Siglec10^−^CD68^−^CD14^+^ myeloid cell population. Herein, only trends of higher accumulation were observed, with a significant exception in the comparison of MNGs over GBMs (Figure 4H). Collectively, an accumulation of Siglec10^+^CD68^+^CD14^+^ was specific to GBM samples, whereas an increase in Siglec10^+^CD68^−^CD14^+^ myeloid cells was characteristic of gliomas in general.

### 3.5 GBMs Show Characteristic Accumulations of CX3CR1^+^Siglec10^+^ Macrophages

Despite GBMs showing an enhanced accumulation of Siglec10^+^CD68^+^CD14^+^ TAMs among CD45^+^ hematopoietic cells in their tumor environment, we still detected many events in MTS to overlap with GBM frequency levels (see Figure 4G, left diagram). To minimize that overlap and define an almost unique GBM‐specific macrophage population, we chose CX3CR1 as an additional marker to characterize TAMs. CX3CR1 in the CNS was originally established as a characteristic marker for microglia, but it also guides peripheral immune cells to CNS structures [39, 40]. Interestingly, it is strongly downregulated on myeloid cells in the microenvironment of CNS metastases [41].

**FIGURE 5:**
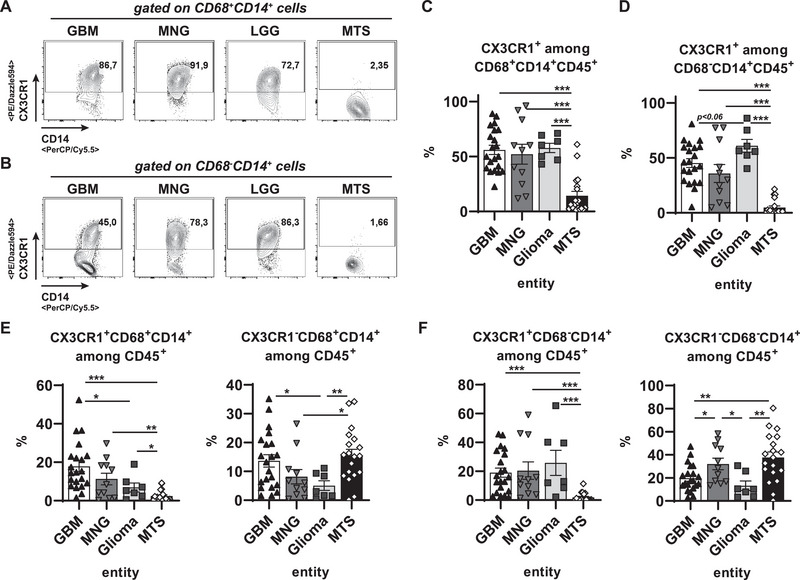
Preferential accumulation of CX3CR1^+^ macrophages in GBM tumor environment. Tumor samples of the indicated entities (GBM: *n* = 21; MNG: *n* = 12; Glioma: *n* = 7; MTS: *n* = 20) were digested, and single cells were stained with antibodies against CD45, CD14, CD68, and CX3CR1. (A, B) Representative dot plots were pregated on living CD68^+^CD14^+^CD45^+^ (A) or CD68^−^CD14^+^CD45^+^ (B) hematopoietic cells. (C, D) Frequencies of CX3CR1 expression among CD68^+^CD14^+^ (C) or CD68^−^CD14^+^ (D) were summarized among living CD45^+^ hematopoietic cells. (E) CX3CR1^+^ (left) or CX3CR1^−^ (right) CD68^+^CD14^+^ cell frequencies among all living CD45^+^ hematopoietic cells were summarized for the indicated entities. (H) CX3CR1^+^ (left) or CX3CR1^−^ (right) CD68^−^CD14^+^ cell frequencies among all living CD45^+^ hematopoietic cells were summarized for the indicated entities. Statistics: ^*^ *p* < 0.05, ^**^ *p* < 0.01, ^***^ *p* < 0.001.

Confirming these observations in our experiments, we could clearly distinguish brain MTSs from primary brain tumor entities via a strongly reduced expression of CX3CR1 among CD68^+^CD14^+^ TAMs (Figure 5A,C) and among CD68^−^CD14^+^ monocytes (Figure 5B,D). Among primary CNS tumor entities, we could hardly detect any differences in CX3CR1 expression frequencies within these subpopulations (Figure 5A–D). In total frequency levels among CD45^+^ hematopoietic cells of the tumor environment, GBM not only accumulated more CX3CR1^+^CD68^+^CD14^+^ TAMs than MTS, but also than non‐GBM‐gliomas (Figure 5E, left diagram), whereas the frequencies of CX3CR1^−^CD68^+^CD14^+^ TAMs appeared comparable between GBMs and MTS (Figure 5E, right diagram). While all investigated primary CNS tumor entities showed comparable ratios of CX3CR1^+^CD68^−^CD14^+^ monocytes, this population was virtually absent among CD45^+^ hematopoietic cells of brain MTS (Figure 5F, left diagram). However, the frequencies of CX3CR1^−^CD68^−^CD14^+^ monocytes appeared comparable among all brain tumor entities, with non‐GBM‐gliomas accumulating the lowest levels (Figure 5F, right diagram). Collectively, CX3CR1^+^ myeloid cells were most strongly increased in primary brain tumor environments compared with those of secondary brain tumor entities with peripheral primary origin.

**FIGURE 6:**
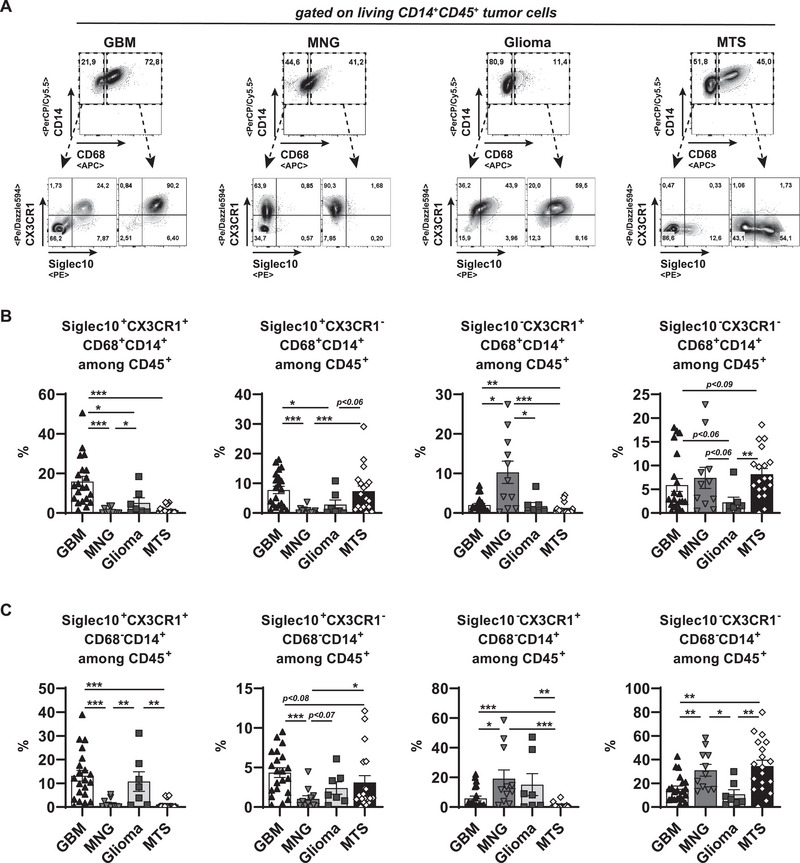
Preferential accumulation of Siglec10^+^CX3CR1^+^ macrophages in GBM microenvironment. Tumor samples of the indicated entities (GBM: *n* = 21; MNG: *n* = 12; Glioma: *n* = 7; MTS: *n* = 20) were analyzed for CD45, CD14, CD68, Siglec10, and CX3CR1 frequencies. (A) Representative gating strategy is shown. (B, C) Indicated Siglec10/CX3CR1 subpopulations of CD68^+^CD14^+^ (B) or CD68^−^CD14^+^ (C) among CD45^+^ cells are summarized. Statistics: ^*^ *p* < 0.05, ^**^ *p* < 0.01, ^***^ *p* < 0.001.

To define distinct myeloid immune subsets in brain tumors, we combined Siglec10 and CX3CR1 in our analysis. Confirming previous results ([41] and Figure 5), on immunohistologically stained tissue sections of the various tumor entities, we could demonstrate that Siglec10 and CX3CR1 colocalized on cellular levels in all gliomas, whereas a Siglec10 signal was absent in meningiomas, and MTS did not show any signal for CX3CR1 staining (Figure S9). A strong accumulation of Siglec10^+^CX3CR1^+^CD68^+^ TAMs was characteristic of GBMs and was significantly higher than that of non‐GBM‐glioma (Figure 6A). Notably, this population was virtually absent from MNGs and MTS (Figure 6A). Both GBMs and MTS showed an enhanced accumulation of Siglec10^+^CX3CR1^−^CD68^+^ TAMs (Figure 6A). Conversely, in MNGs, the frequencies of Siglec10^−^CX3CR1^+^CD68^+^ TAMs were selectively enhanced (Figure 6A). Siglec10^−^CX3CR1^−^CD68^+^ TAMs were comparably represented in all entities with a trend to lower frequencies in non‐GBM‐gliomas (Figure 6A). In contrast to the microglia/macrophage population, Siglec10^+^CX3CR1^+^CD68^−^CD14^+^ monocytes were overrepresented in all gliomas, including GBMs, whereas no entity‐specific accumulation of any other CD68^−^CD14^+^ monocyte populations was detected (Figure 6B). Therefore, in GBM tumors, Siglec10^+^CX3CR1^+^ TAMs potentially represent an indicator cell population.

### 3.6 GBM Lipid TME Differs From That of Other Entities

**FIGURE 7:**
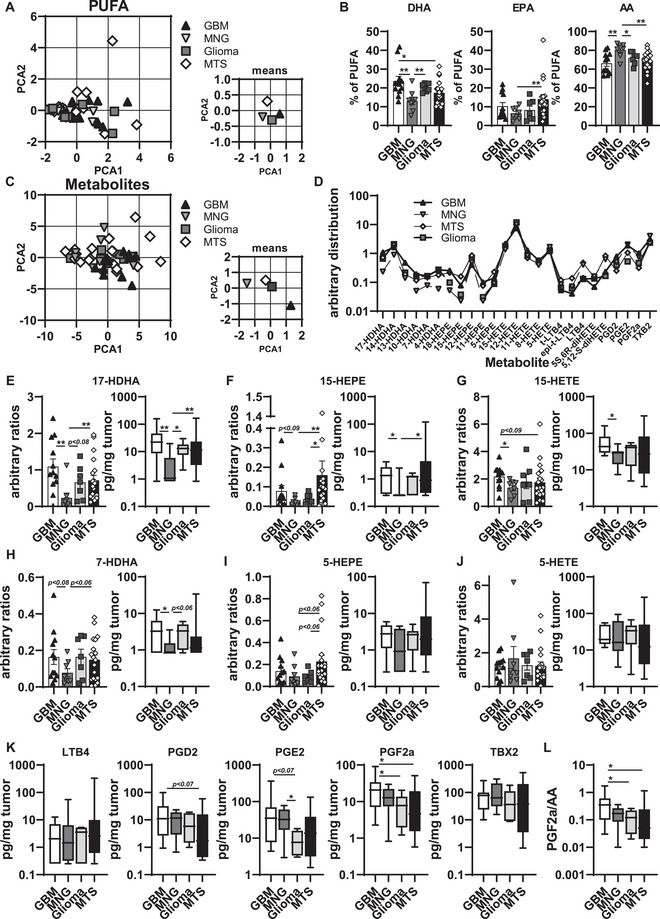
PUFA and their metabolism in GBM microenvironments compared with other tumor entities. PUFAs and LM metabolites were analyzed in various CNS tumor entities (GBM: *n* = 13; MNG: *n* = 8; Glioma: *n* = 8; MTS: *n* = 7). (A, B) PCA was calculated for absolute amounts of PUFAs for each entity (A), and the relative distribution compared with the absolute amount of PUFAs (sum of AA, EPA, and DHA) is shown for each tumor entity (B). (C) PCA of PUFA metabolites was calculated for each entity. (D–L) Relative distribution of all analyzed PUFA metabolites was calculated as arbitrary ratios and compared.

GBM tumor cells possess an altered lipid metabolism to adapt to limited nutrients and to be independent from exogenous lipid supply [42]. Lipid metabolites in the TME regulate the differentiation and polarization of macrophages and thereby support a local immunosuppression [43]. Here, we applied targeted liquid chromatography–tandem mass spectrometry–based metabololipidomics to analyze and quantify specific lipid mediator (LM) profiles in tumor entities. When we performed a principal component analysis (PCA) of the measured free polyunsaturated fatty acids (PUFA), namely AA, EPA, and DHA within the tumor entities, the overall differences between the various tumor samples were very small (Figure 7A). To overcome the high variation in the absolute PUFA content between different patient samples, we calculated the relative distribution of selected PUFAs, which revealed a higher representation of AA in MNG samples, a higher DHA representation in GBM samples, and a higher EPA representation in MTS samples (Figure 7B). When we performed a PCA of the measured LMs, we could identify that the MTS and MNG clusters differ from the cluster of GBM and non‐GBM glioma samples, reflecting the origin of the tumors (Figure 7C). To compare the metabolic patterns of the different tumor entities, we compared the arbitrary distributions of all LMs (Figure 7D). The MNG samples showed an underrepresentation of DHA and EPA metabolites, whereas non‐GBM gliomas had only underrepresented EPA metabolites (Figure 7D). Interestingly, all entities comprised AA metabolites at comparable ratios, whereas COX‐dependent end products differed (Figure 7D). In line with reduced amounts of DHA in comparison to other entities. MNGs had the lowest amounts of 5‐ and 15‐LOX products, namely 7‐ and 17‐HDHA (Figure 7E,H). In contrast to this, the high levels of AA did not translate into higher amounts of the 15‐LOX product 15‐HETE in MNGs (Figure 7B,G), whereas GBMs showed higher amounts of 15‐HETE despite lower AA products from AA (Figure 7B,G). No differences could be observed between the 5‐LOX products 5‐HEPE (from EPA), 5‐HETE, or LTB4 (both from AA) of the different entities (Figure 7I–K). In line with the varying representation of COX‐derived AA metabolites (see Figure 7D), we detected entity‐dependent alterations of the amount of several prostaglandins (Figure 7K). Throughout all comparisons, GBMs had very high amounts of PGD_2_, PGE_2_, and PGF_2a_, albeit MNGs showed high amounts as well (Figure 7K). When we related the amounts of PGF2a to those of AA, PGF_2a_ remained high in GBMs, whereas in MNGs it decreased to levels comparable to the other tested tumor entities. However, when we stimulated monocytes with PGD_2_, PGE_2_, and PGF_2a_ in concentrations previously shown to modulate monocytes/ macrophages [44], we did not detect any upregulation of Siglec10 expression (Figure S10). Collectively, we observed a variation in the amounts of PUFA metabolites between different CNS tumor entities and could detect increased levels of COX‐dependent prostaglandins in GBMs, most significant in PGF_2a_.

### 3.7 Sigelc10^+^CX3CR1^+^ TAMs Accumulate in the PPIX^+^ GBM Tumor Core Area

**FIGURE 8:**
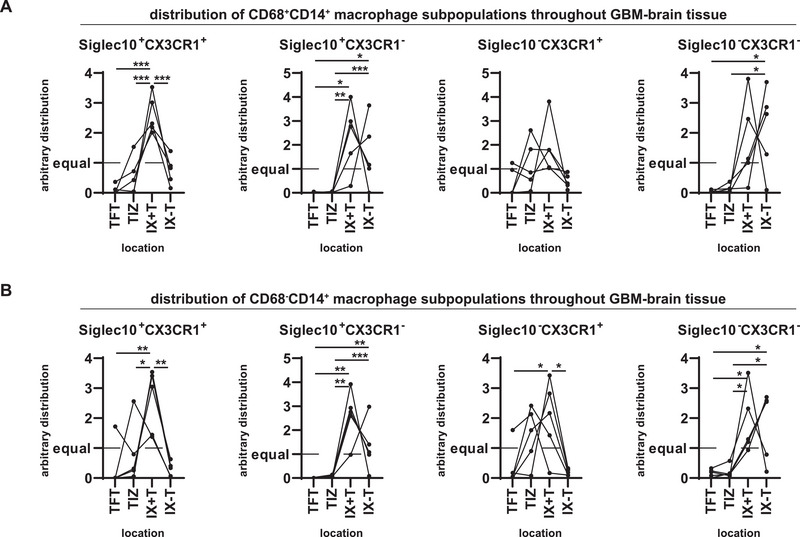
Siglec10^+^CX3CR1^+^ TAMs accumulate in the active GBM tumor core. To allocate the distribution of the analyzed subpopulations, all frequencies of a particular population in the distinct samples were related to their average frequency in different regions of each separate tumor. (A, B) Indicated Siglec10/CX3CR1 subpopulations of CD68^+^CD14^+^ cells (A) or CD68^−^CD14^+^ cells (B) among CD45^+^ cells in the indicated part of the TME were analyzed. Statistics: ^*^ *p* < 0.05, ^**^ *p* < 0.01, ^***^ *p* < 0.001.

When we investigated the distribution throughout the tumor environment of GBMs as guided by intrasurgical PPIX fluorescence, we could clearly see that Siglec10^+^CX3CR1^+^ and Siglec10^+^CX3CR1^−^ monocytes and macrophages preferentially accumulated in the central tumor areas, with bias toward areas of PPIX^+^ tumor cells (Figure 8A,B). In contrast to this, Siglec10^−^CX3CR1^−^ macrophages preferentially gathered in PPIX^−^ central tumor areas (Figure 8A,B). Interestingly, Siglec10^−^CX3CR1^+^ monocytes as well as macrophages have the most balanced distribution without any preferential localization, reflecting a general CNS tissue tropism (Figure 8A,B). Collectively, in active PPIX^+^ GBM areas, Siglec10‐expressing myeloid cells were most prominent, while populations of CX3CR1‐expressing subsets were distributed throughout all tumor areas, and Siglec10^−^CX3CR1^−^ TAMs were overrepresented in the PPIX^−^ necrotic GBM core (Figure 8A,B).

## 4 Discussion

In this study, we compared the myeloid cell subsets among various tumor entities and identified a GBM‐specific macrophage subpopulation, which expresses both Siglec10 and CX3CR3. In contrast to the other investigated intracranial neoplasias, the presence of GBM specifically correlated with a TME of relatively high DHA, 17‐HDHA, and 7‐HDHA levels, and high levels of AA‐derived COX products, that is, PGE_2_ and PGF_2a_.

The tumor microenvironment is a very complex milieu that is a result of multiple factors, among them a lack of space, nutrients, and oxygen due to tumor growth, a dynamic communication between cancer cells and local tissue or immune cells, and an alteration of local mediators due to abnormal tumor cell metabolism. These environmental changes, plus intrinsic genetic alterations of tumor cells per se, complicate analyses based on the sole comparison of GBM tissues with tumor‐free brain tissues. Maybe one of the most promising approaches using such a strategy has been the analyses of differential gene expressions between tumor‐free tissues and GBM‐derived tissues by either bulk sequencing or single‐cell sequencing [8, 45, 46]. Based on these findings, diverging characteristics of different GBMs resulted in separate subclassifications like classical, proneural, neural, or mesenchymal subtypes [45, 46]. Associated with these subtypes, altering compositions of macrophage populations have been documented [8]. However, none of these findings resulted in any benefit for the development of GBM therapies, yet [4]. To distillate GBM‐specific characteristics of macrophage populations from general tumor characteristics, we combined two separate approaches. First, we compared the GBM sample data with prominent primary and secondary brain tumor entities, but not with tumor‐free tissues. We chose meningiomas (MNGs) as the most frequent primary brain tumor [47]. MNGs develop from arachnoidal cap cells and grow slowly, but they ultimately smother surrounding tissue, eventually invade adjacent bone structures, and cause stress‐related changes in the TME [48]. To integrate malign tumors of peripheral origin, we analyzed fast‐growing metastatic brain tumors. They grow in an encapsulated, but often multifocal fashion [49] and due to their peripheral origin, the modification of the tissue environment is potentially different from primary tumor entities [50]. Additionally, we examined non‐GBM gliomas, which often share an origin related to GBMs. On average, non‐GBM gliomas are less aggressive than GBMs and are characterized by varying genetic alterations [47]. By this comparative strategy, including various other tumor entities, we filter out most of the common tumor‐related changes and potentially uncover GBM‐specific changes. Our second approach was the use of protoporphyrine IX (PPIX) signals to visually landmark GBM tumor samples. This helped us to minimize variations arising from heterogeneous consistency of tumor areas, like necrotic cell core areas or invasion zones with low tumor cell presence. Of note, PPIX is not restricted to tumor cells per se, but it is potentially accumulated in biologically active cells of tumor core areas [32]. Based on the visualization of the PPIX in combination with the surgeon's orientation in situ, we selected GBM tumor areas including tumor‐free entrance tissue (PPIX‐), tumor invasion zone (PPIX‐ tissue close to the tumor core), PPIX+ tumor core (active tumor cell area), and PPIX‐tumor core (necrotic zone).

Tumor cells from GBMs have been classified into four different subtypes according to Verhaak nomenclature [46], which have been substantiated by a lack of IDH mutation and varying MGMT promoter methylation statuses [51]. In contrast to the genetic alterations and varying phenotypes of the GBM cancer cells, leukocytes originate from a common (peripheral) source and are individually shaped by the TME microenvironment upon infiltration. Considering the almost similar prognoses and histological features of all these different GBM subtypes, we analyzed the composition of leukocyte populations to find a common GBM‐related immunological feature, which might be associated with GBM specifically. Except for granulocytes and other myeloid cells, we could not detect any preferential site of immune cell accumulation. Granulocytes had a slight preference toward localization in necrotic PPIX‐ GBM core areas (IX‐T), whereas myeloid cells preferentially accumulated in active PPIX+ GBM core areas (IX+T). That is in line with earlier observations showing that during glioma development, myeloid cells are recruited to the TME and during initial tumor development, they preferentially locate in the perivascular areas [17, 52]. Moreover, in established primary and metastasizing CNS tumors, infiltrating macrophage populations of different ontogeneses have been observed [53, 54]. Tumor‐associated microglia‐like macrophages (TAM‐MG) and monocyte‐derived macrophages (TAM‐MDM) are considered two major subpopulations, but a functional distinction has not been made yet [55, 56, 57]. Nevertheless, these macrophages exhibit a population‐specific spatial distribution, wherein TAM‐MG mainly occur in the tumor periphery and TAM‐MDM within the tumor core [15, 58, 59]. Of note, all these reports are hampered by discrepancies in marker expression used to identify both major populations. The discrimination used in mice based on CD45 expression levels does not apply to human situations [52]. The key TAM‐MDM markers CCR2 and Ly6C are downregulated in glioma environments [60], whereas the expression of one of the microglia markers, P2RY12, is reduced in gliomas or other disease‐associated settings [61, 62]. Similar changes are described for microglia‐associated Sall1 and Tmem119 expression [61, 62]. Most of the TAM‐MDM that originate from infiltrating monocyte precursors are reprogrammed in the TME, resulting in populations with altered gene expression, in particular, the microglia‐associated CX3CR1 is upregulated on TAM‐MDM [52, 56]. Collectively, no reliable marker exists to discriminate between CNS macrophages and microglia in disease settings. To identify general myeloid cells, we chose the marker CD14, and subsequently, we discriminated between CD68^+^ macrophages/microglia and CD68^−^ monocytes [63]. Of note, in our datasets, we cannot differentiate between macrophages and microglia subsets, and therefore, all conclusions drawn should consider both macrophage populations.

Using the PPIX‐guided visual identification of GBM tumor areas, we could confirm and extend previous murine and human data on immune cell distribution in GBMs [64, 65] by flow cytometry. While in tumor‐free tissue, CD14^+^ myeloid cells are represented at levels comparable to other immune cell subpopulations, approximately 50% of the CD45^+^ cells in that area were negative for all chosen markers (data not shown) and most likely represent inactive, resident CD14^−^ microglia [66, 67]. We defined the PPIX‐negative tissue around the tumor core as the tumor infiltration zone (TIZ). Notably, the frequency of CD14^+^ myeloid cells increased in the TIZ and cumulated in the PPIX‐positive tumor core (IX+T), representing almost 50% of all hematopoietic cells. In those IX + T areas, we also detected the highest accumulation of CD68^+^CD14^+^ macrophages. In contrast to that, but in line with previous reports [68], granulocytes represented the most prominent leukocyte population in the PPIX‐negative necrotic tumor core area (IX‐T). When we characterized the CD14^+^ myeloid cells throughout the TME, we detected a preferential accumulation of Siglec10^+^ macrophages in the IX+T areas irrespective of their CX3CR1 expression status. While CX3CR1^+^Siglec10^−^ macrophages appeared equally distributed throughout all GBM tumor areas, CX3CR1^−^Siglec10^−^ macrophages preferentially accumulated in the necrotic areas. Given the role of CX3CR1 in tropism throughout brain tissue [69], CX3CR1^+^Siglec10^−^ macrophages patrol through the tissue, but might not have yet interacted with tumor cells. These tissue‐remodeling macrophages can at least in part arise from classical monocytes in the circulation and, upon entering CNS tissues, might depend on CX3CR1 expression for survival [70]. During glioma progression in genetically engineered mice, it was observed that tumor‐associated microglia are continuously replaced by monocyte‐derived macrophages [71]. Whether this is translatable to the human environment remains to be proven. GBM necrotic areas originate from obstructed blood vessels that initially supplied the tumor [72]. Thus, Siglec10^−^CX3CR1^−^ monocytes and macrophages might have entered that area via blood vessels and participate in local tissue remodeling and pseudovascularization [72]. Even though the distribution of monocyte subpopulations mainly resembles that of macrophages, Siglec10^+^CX3CR1^−^ monocytes accumulated more strongly in the necrotic tumor area than their macrophage counterparts. This suggests that these monocytes represent precursor cells for tumor‐resident TAMs, which, upon arrival at the tumor area, become activated and modulated by the TME. While CX3CR1‐negative monocytes preferentially accumulated in the IX‐T necrotic center, CX3CR1‐expressing monocytes did not, and Siglec10^+^CX3CR1^+^ monocytes could be detected predominantly in the IX+T tumor core. Collectively, Siglec10^+^CX3CR1^+^ macrophages and monocytes accumulated in GBM tumor core areas with high PPIX signals. As PPIX signals point toward an enhanced cellular activity [32], Siglec10^+^CX3CR1^+^ TAMs are a potential result of infiltrating monocytes modulated by local tumor cells. Of note, not only monocytes or macrophages express CD14 as a surface marker, but also type 3 dendritic cells (DC3) expressing CD14 can be detected in tumors, albeit at low frequencies [73]. Thus, the conclusion generally drawn for CD68^−^CD14^+^ in this manuscript has to be refined for the potential presence of DC in future studies. However, we could remodel an interaction between monocytes and cocultured primary GBM tumor cells in an *in vitro* setting, which demonstrated a GBM tumor‐mediated upregulation of monocytic Siglec10. When we compared the appearance of Siglec10^+^CX3CR1^+^ TAMs throughout various tumor entities, we detected a highly specific enrichment within GBMs. However, few but significantly fewer Siglec10^+^CX3CR1^+^TAMs were also detected in non‐GBM‐gliomas. Similarly, reduced frequencies of Siglec10^+^CX3CR1^−^ TAMs in non‐GBM‐gliomas suggest that the glioma TME‐enriched Siglec10^+^ macrophages are at a level correlating with malign phenotypes. Supporting this, Siglec10^+^CX3CR1^−^ TAMs predominated in both highly malignant tumor entities, GBMs and MTS. Since MTS macrophages do not express CX3CR1 [74], the combination of CX3CR1 with Siglec10 might specifically identify macrophages that support the highly diffuse and infiltrative nature of GBM tumor cells into healthy surrounding tissues. CX3CR1 is involved with glial‐neuronal crosstalk and guidance of immune cells within the brain environment [69, 75].

This is underlined by the observation that brain MTSs lacking CX3CR1‐expressing macrophages exhibit less diffuse growth. Supporting this, patients with brain MTS accompanied by an inactive primary tumor disease have a much longer overall survival [76]. Of note, while MNGs showed comparable frequencies of overall macrophages and monocytes, hardly any Siglec10‐expressing myeloid cells could be detected, and thus, MNGs display the highest accumulation of Siglec10^−^CX3CR1^+^ TAMs. Of note, CX3CR1 expression in brain tumors is not associated with changes in overall survival [77]. Therefore, we could exclude spatial stress as a criterion for Siglec10^+^ TAM generation but would rather correlate a tumor cell‐mediated mechanism. Reflecting this, we could not induce Siglec10 expression on monocytes by the presence of MNG tumor cells in vitro.

Despite metastases sharing the origin with their primary source tumor, they considerably diverge from it on the transcript level upon establishment in brain tissue [78]. In contrast to this, intracranial metastases appeared to be highly homogenous compared with extracranial metastases, even when spatially and temporally separated [78]. This suggests that the brain environment is an important driver of specific tumor characteristics and that an additional alteration of the TME might be specific for distinct tumor entities. DHA is predominant in brain tissue, and by analyzing PUFA levels and their metabolites, we detected a significant predominance of DHA in gliomas, whereas MNGs showed an AA predominance. This suggests that the predominance of specific PUFAs in the TME could be correlated with the particular tumor entities and their levels of Siglec10^+^CX3CR1^+^ TAMs. Higher concentrations of EPA or DHA at the expense of AA have been associated with a suppressive effect on proinflammatory macrophage activation and the induction of a tissue‐remodeling macrophage phenotype [79, 80]. Reflecting its least malign phenotype, MNGs show a strong predominance of the proinflammatory AA. However, the corresponding 15‐LOX products of this PUFA were not enriched in those TMEs, which points to a low activity of 15‐LOX. Concomitantly, the presence of Siglec10^+^ macrophages is the lowest in MNGs, whereas the presence of Siglec10^−^CX3CR1^+^ macrophages is highest among all investigated tumor entities. Vice versa, an enhanced amount of 17‐HDHA correlated with the enhanced Siglec10 induction in malignant tumor entities, and an enhanced presence of 7‐HDHA correlated with enhanced Siglec10^+^CX3CR1^+^ myeloid cells. Uhlen et al. [81] show that high‐grade gliomas do not express 5‐LOX mRNA. However, levels of 5‐LOX products were decreased in GBMs. Although we detected comparably high amounts of 15‐LOX products in malign tumor entities, this was not translated into increased 5‐LOX products, pointing to decreased 5‐LOX activity. Taking it together, the presence of 7‐HDHA might promote the accumulation of Siglec10^+^ CX3CR1^+^ myeloid cells in gliomas, which in GBMs would be matured into Siglec10^+^CX3CR1^+^ TAMs by the presence of enhanced amounts of COX products in the local TME. However, we could rule out a direct effect of prostaglandins PGD_2_, PGE_2_, and PGF_2a_.

## 5 Conclusion

In our study, we presented a Siglec10^+^CX3CR1^+^ TAM population, which was not only unique to primary brain tumors but also characteristic of GBMs. We further propose that the local lipid microenvironment supports these specific macrophages. Based on our results, detection of Siglec10^+^CX3CR1^+^ TAMs might not only be of diagnostic value, but also potentially offer a new target for therapeutic strategies. However, a functional characterization is required, and a direct association between Siglec10^+^CX3CR1^+^ TAMs and a specific local lipid mediator milieu remains to be proven.

## 6 Limitations

In our study, we compared selected entities, particularly the most prominent CNS tumor entities. These analyses need to be expanded to other tumor entities of lesser incidence. The interpatient variations of lipid mediator levels and cell compositions within each tumor entity group are very high, and therefore, more data need to be generated to enable a stronger statistical groundwork for the development of therapeutic strategies or clinical applications. Our hypothesis and findings are based on retrospective analyses of the acquired data. Prospective studies will be needed to verify the prognostic value of Siglec10^+^CX3CR1^+^ TAMs for GBM diagnosis.

## Author Contributions

N.A. and C.S. designed the concept, N.A. directed the project, drafted and wrote the manuscript, L.G. and N.A. performed experiments and analyzed data, P.M.J. analyzed lipid mediators, S.S. generated the histological data, P.M.J., C.S., O.W., and P.B. gave valuable input and critically revised the manuscript, C.S., P.B., N.D., and the OncoSurgJena team collected samples as well as performed PPIX‐guided sampling. **OncoSurgJena team (**Department of Neurosurgery, Jena University Hospital, Friedrich Schiller University, Am Klinikum 1, D‐07747, Jena, Germany): PD Dr. med. Falko Schwarz, FEBNS, MBA; Dr. med. Nazeer Aboud, FEBNS; Dr. med. Ramazan Dalkilic; Dr. med. Sergio A. Calero Martinez; Dr. med. Katharina Klumbies; M.Sc. Aaron Lawson McLean.

## Ethics Statement

All samples were analyzed in accordance with the Declaration of Helsinki. Tissue and data analysis were approved by the institutional ethics committees of the Jena University Hospital and the respective data protection commissioner (registration number 2019‐1400). All patients gave their written informed consent. Leukopaks (buffy coats) were received from the Institute of Clinical Transfusion Medicine Jena GmbH (IKTJ). All donors have been informed before the full blood donation and gave their written consent to the use of the blood components. The distribution license was authorized by the Thuringian State Office for Consumer Protection (DE_TH_01H_WDA_2023_11*).

## Conflicts of Interest

All authors declare no conflicts of interest.

## Supporting information




**Supporting File**: eji70180‐sup‐0001‐SuppMat.pdf.

## Data Availability

The authors confirm that all relevant data supporting the findings of this study are included within the paper and its supplementary information files. Raw data can be assessed from the corresponding author upon reasonable request.
