# Supplementary material for: Accumulation of Siglec10+CX3CR1+ Macrophages in the Tumor Microenvironment of Glioblastomas
Source: Eur J Immunol. 2026 Apr 20;56:e70180. doi: 10.1002/eji.70180 (PMC13093235; doi:10.1002/eji.70180)
Supplement: Supplementary file 1 — Supporting File: eji70180‐sup‐0001‐SuppMat.pdf. [file EJI-56-e70180-s001.pdf]

Supplement Figure 1

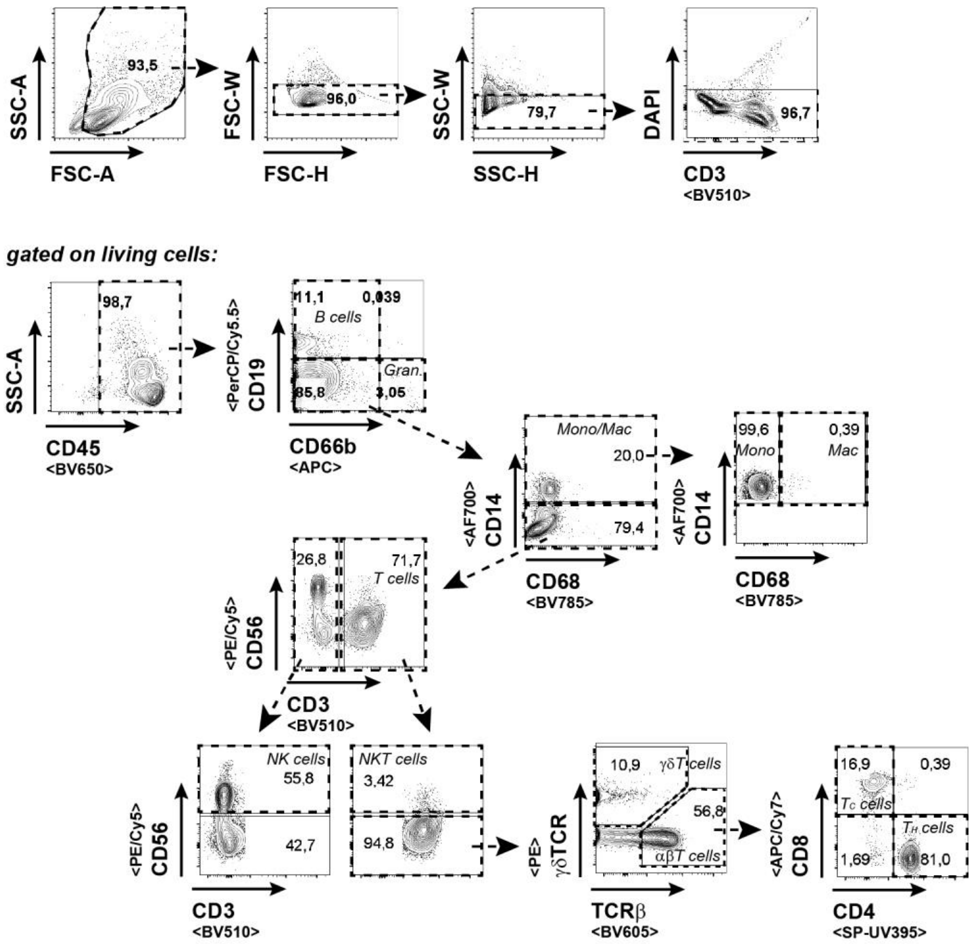

Suppl. Fig. 1 Identification of hematopoietic cells in peripheral blood mononuclear cells (PBMCs): PBMCs were isolated from buffy coats by gradient centrifugation and subsequently stained for FACS analysis. Representative FACS plot showing the identification of living DAPI$^-$ single cell structure. Based in this, further gating strategies identifying CD45$^+$ subpopulations are shown.

Supplement Figure 2

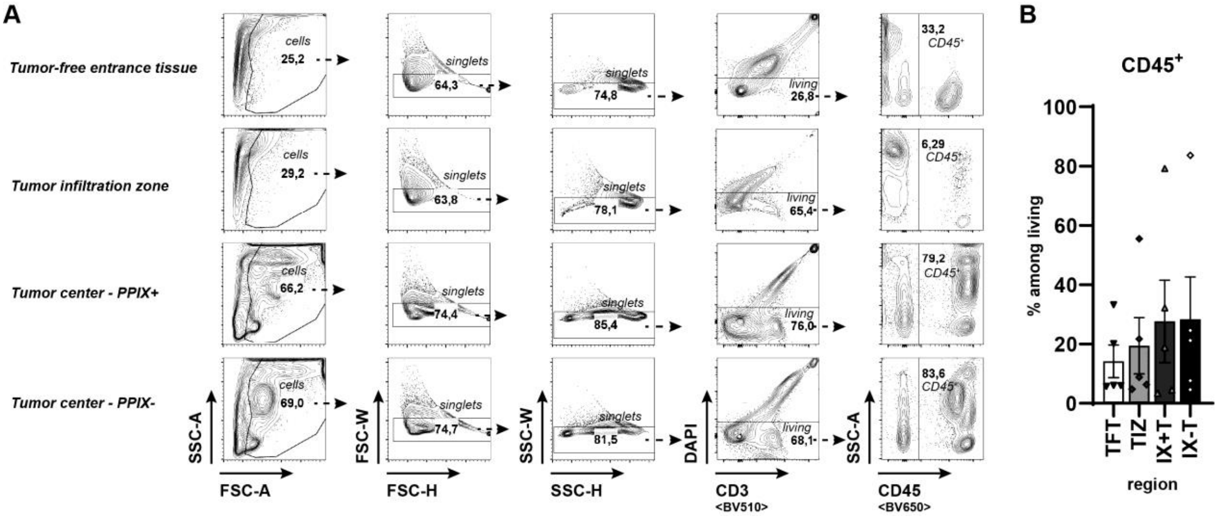

Suppl. Fig. 2 Identification of hematopoietic cells in different tumor regions of GBM patients: A) Representative FACS plot showing the identification of $CD45^+$ cells in living $DAPI^-$ single cell structure. **B)** Summarized data showing $CD45^+$ cell frequencies in different GBM tumor regions. Abbreviations: Tumor-free entrance tissue: TFT; Tumor infiltration zone: TIZ; PPIX+ tumor regions: IX+T; PPIX- tumor regions: IX-T.

Supplement Figure 3

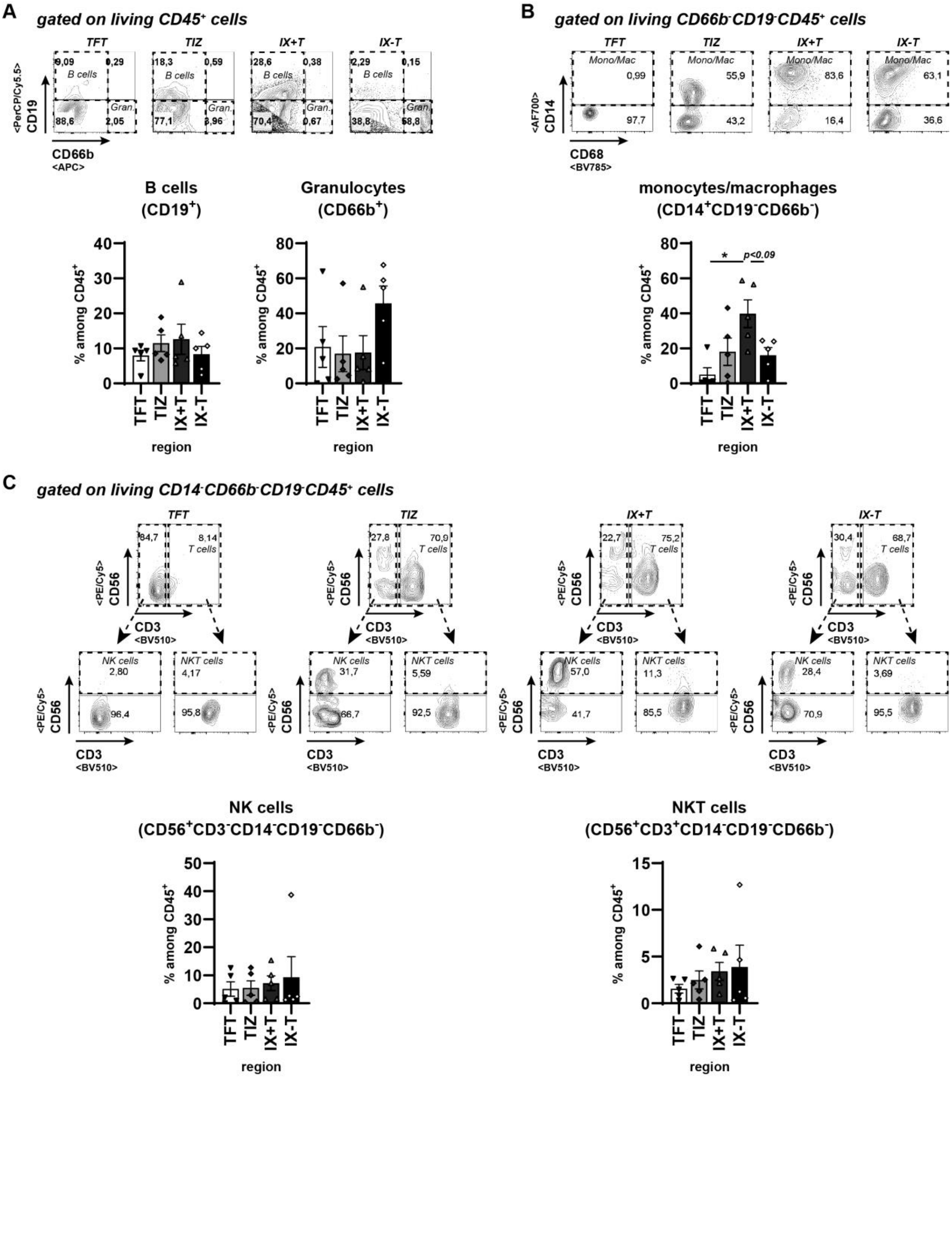

Suppl. Fig. 3: Identification of immune cell subsets in different areas around GBM tumor center. Representative FACS plots are shown above the summarizing diagrams. All analyses were initially gated on living $CD45^+$ hematopoietic cells and frequencies are summarized among that $CD45^+$ compartment. **A-C)** B cells or granulocytes were identified by expression of CD19 or CD66b, respectively (A). Among CD19-CD66b- non-B cell/non-granulocytes, CD14 was analyzed to identify myeloid cells (B). Among CD14-CD19-CD66b- cells, different combinations of the markers CD56 and CD3 were utilized to identify $CD56^+CD3^-$ NK cells and $CD56^+CD3^+$ NKT cells (C).

Supplement Figure 4

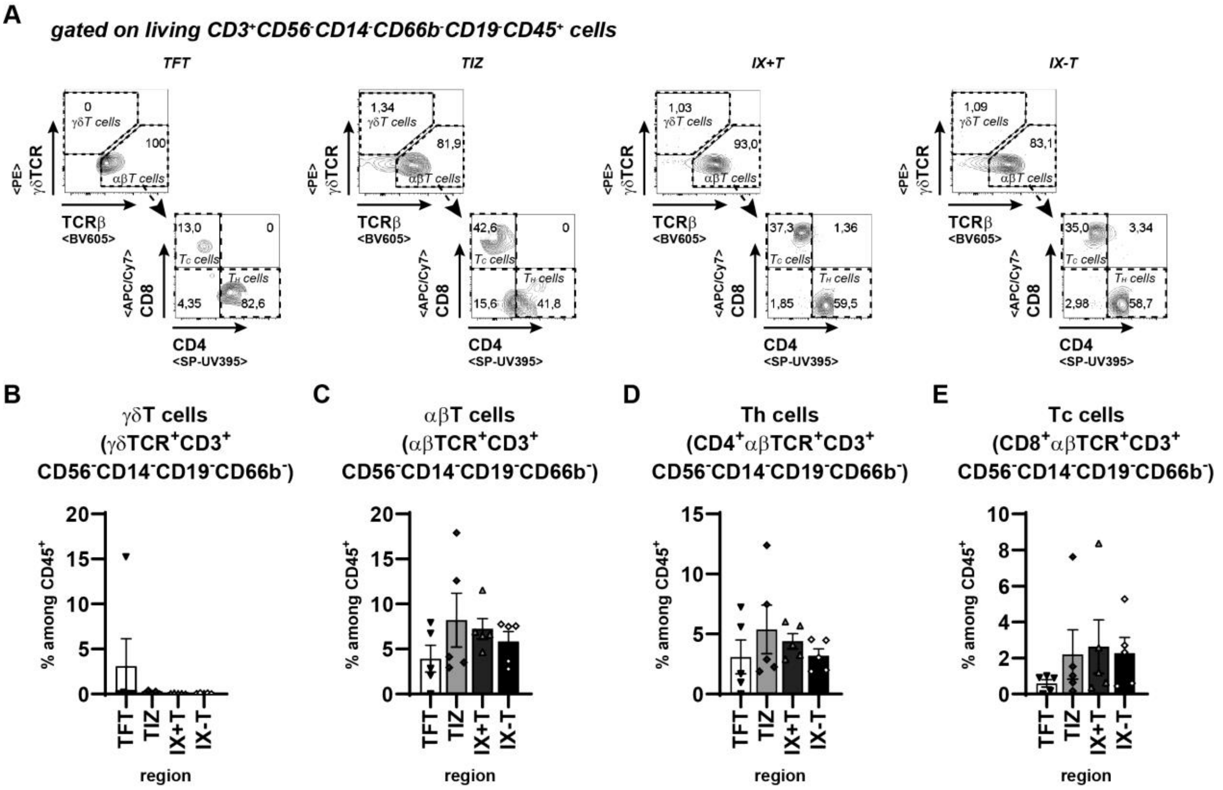

Suppl. Fig. 4: Identification of various T lymphocyte subsets in different areas around GBM tumor center. Representative FACS plots are shown above the summarizing diagrams. All analyses were initially gated on living $CD3^{+}CD56^{-}CD14^{-}CD19^{-}CD66b^{-}CD45^{+}$ hematopoietic cells and frequencies are summarized among the overall $CD45^{+}$ compartment. **A)** γδT cells and αβT cells were identified by expression of γδTCR or TCRβ, respectively. Subsequently T_H and T_C cells were identified by CD4 or CD8 expression, respectively. **B-E)** Frequencies of γδT cells (B), αβT cells (C), T_H cells (D) or T_C cells (E) among $CD45^{+}$ hematopoietic cells are summarized in diagrams.

Supplement Figure 5

A

Gene name	fold change GBM/healthy	GBM	healthy	significance
CD47	1,93	59,75	30,91	n.s.
SIRPA	0,55	69,55	126,24	n.s.
CD24	7,65	33,59	4,39	sign.
SIGLEC10	14	15,72	1,12	sign.
LILRB1	5,78	6,42	1,11	sign.
HLAA	12,5	991,04	79,13	sign.
HLAB	12,3	1002,99	81,79	sign.
HLAC	8,25	596,26	72,25	sign.
CD274	2,67	1,95	0,73	n.s.
CD279	9,33	0,28	0,03	n.s.
		GBM	healthy	
data set size		**163**	**207**	

B

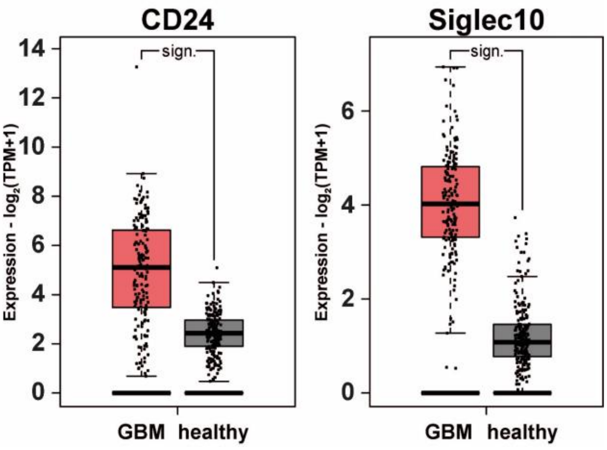

Suppl. Fig 5: The most upregulated “Don’t eat me” signals in GBM is the CD24-Siglec10 signalling axis. A) GEPIA analysis of the TCGA datasets for the indicated “Don’t eat me” signaling partners. **B)** Separate visualization of CD24 or Siglec10 transcripts filtered from the TCGA dataset. All statistics were performed by the GEPIA data base and indicated as significant (sign.) when $P<0.05$ was achieved.

Supplement Figure 6

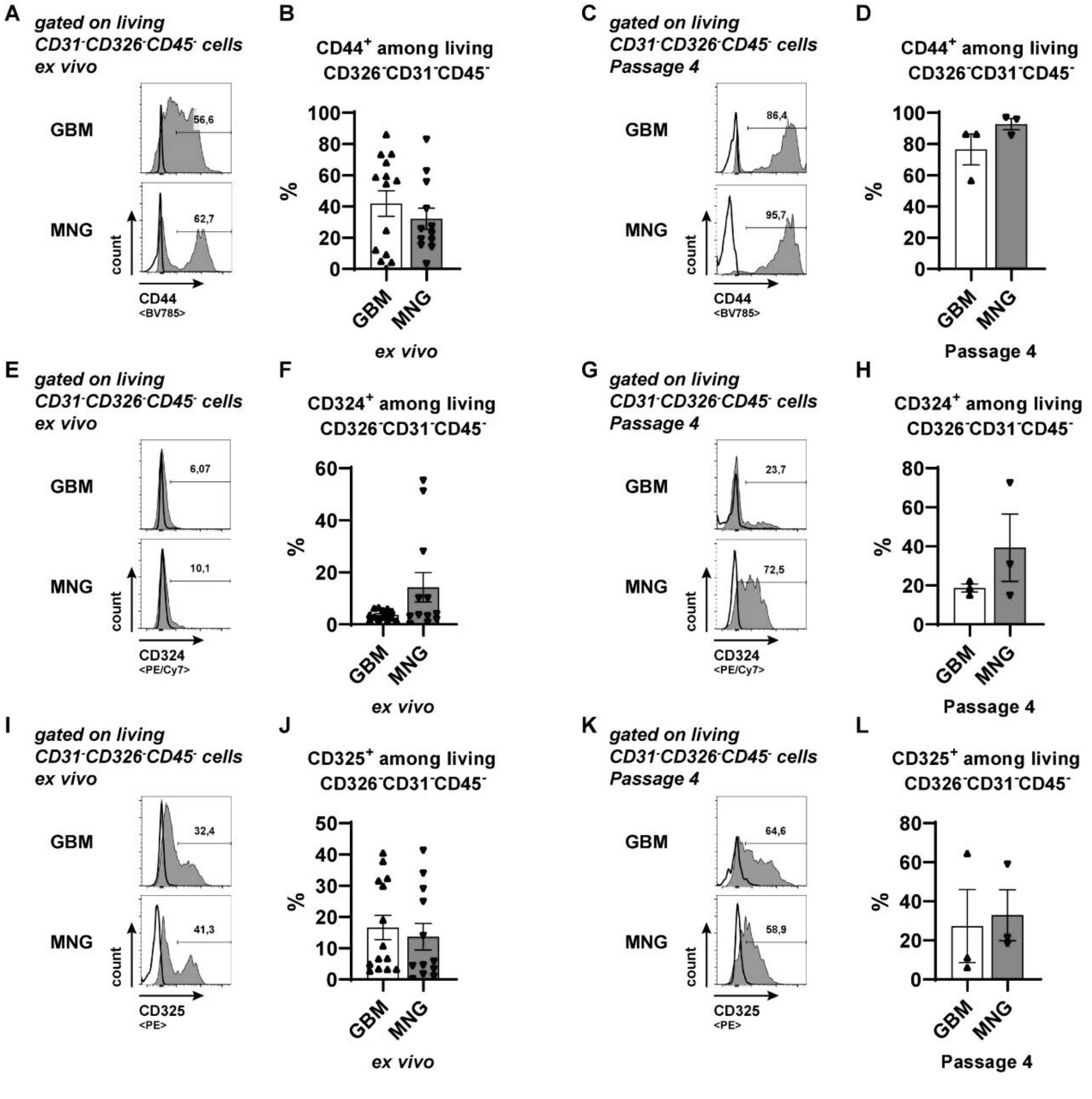

Suppl. Fig. 6: Comparable expression of CD44, CD324 and CD325 among tumor cells in primary cell culture of GBM and MNG cells. Tumor samples of the indicated entities were digested, and single cells were stained with antibodies against CD45, CD31, CD326 and CD44, CD324 or CD325. **A-D)** Representative *ex vivo* signals of CD44 among living $CD31^-CD326^-CD45^-$ cells of the indicated tumor entities (A) with summarized data depicted in the diagram (B) *ex vivo* or when obtained from subsequent primary tumor cell culture at passage 4 (C, D). **E-H)** Representative *ex vivo* signals of CD324 among living $CD31^-CD326^-CD45^-$ cells of the indicated tumor entities (E) with summarized data depicted in the diagram (F) *ex vivo* or when obtained from subsequent primary tumor cell culture at passage 4 (G, H). **I-L)** Representative *ex vivo* signals of CD325 among living $CD31^-CD326^-CD45^-$ cells of the indicated tumor entities (I) with summarized data depicted in the diagram (J) *ex vivo* or when obtained from subsequent primary tumor cell culture at passage 4 (K, L).

Supplement Figure 7

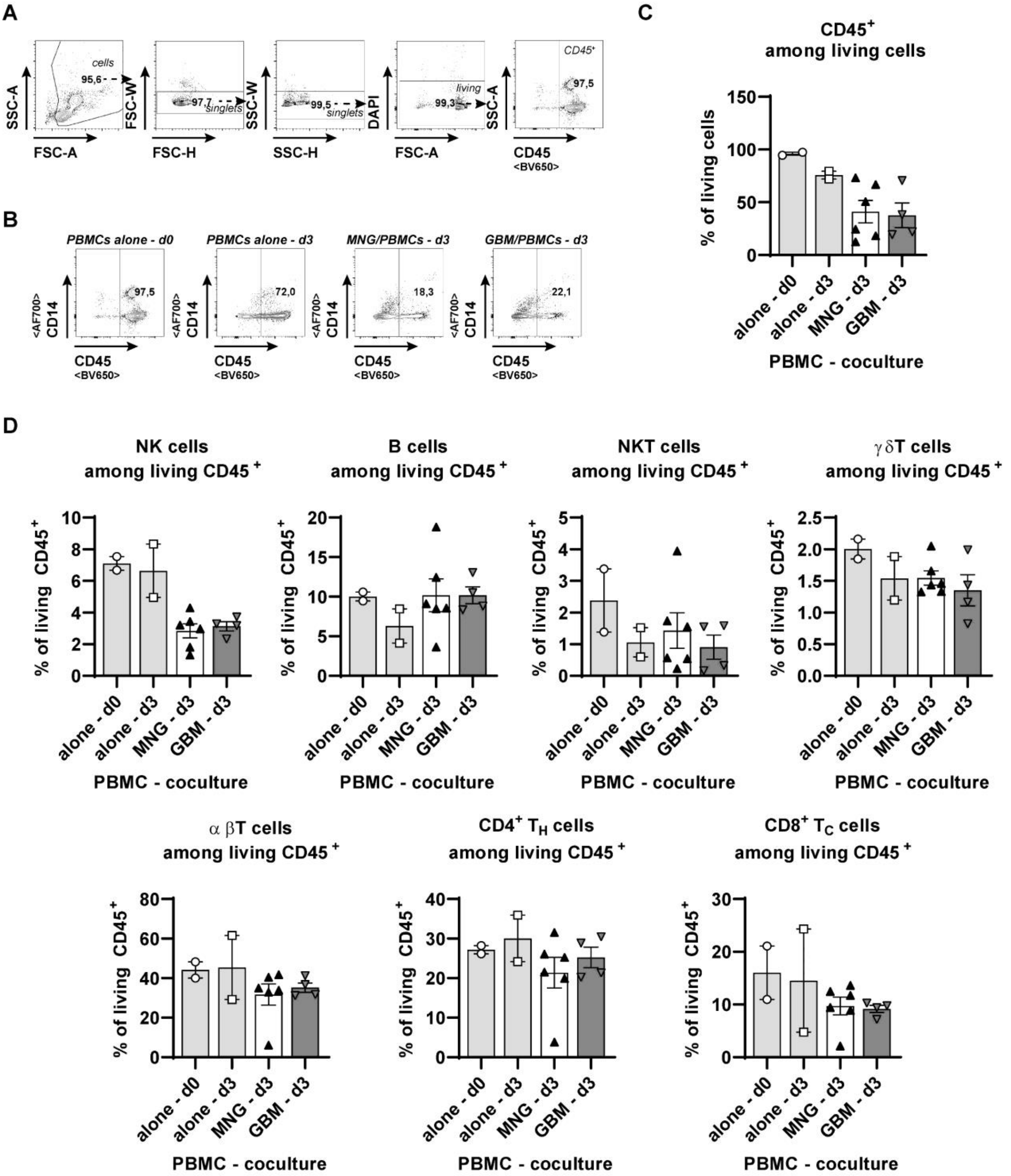

Suppl. Fig. 7: GBM tumor cells do not preferentially maintain specific immune subsets. PBMCs were purified from 2 healthy donors. After 3 days of coculture of the PBMCs with 50,000 primary tumor cells at a 20:1 ratio, all culture cell were detached and analyzed by flowcytometry as described in the Material and Method section or as shown before. **A)** Gating strategy for $CD45^+$ hematopoietic cells in freshly isolated PBMCs. **B)** Summary of $CD45^+$ PBMCs recovered from all cultures as indicated (3 separate MNGs, 2 separate GBMs) after 3 days of culture. **C)** Subsets of immune cells in all indicated conditions were analyzed as described in Material and Method section or as shown before.

Supplement Figure 8

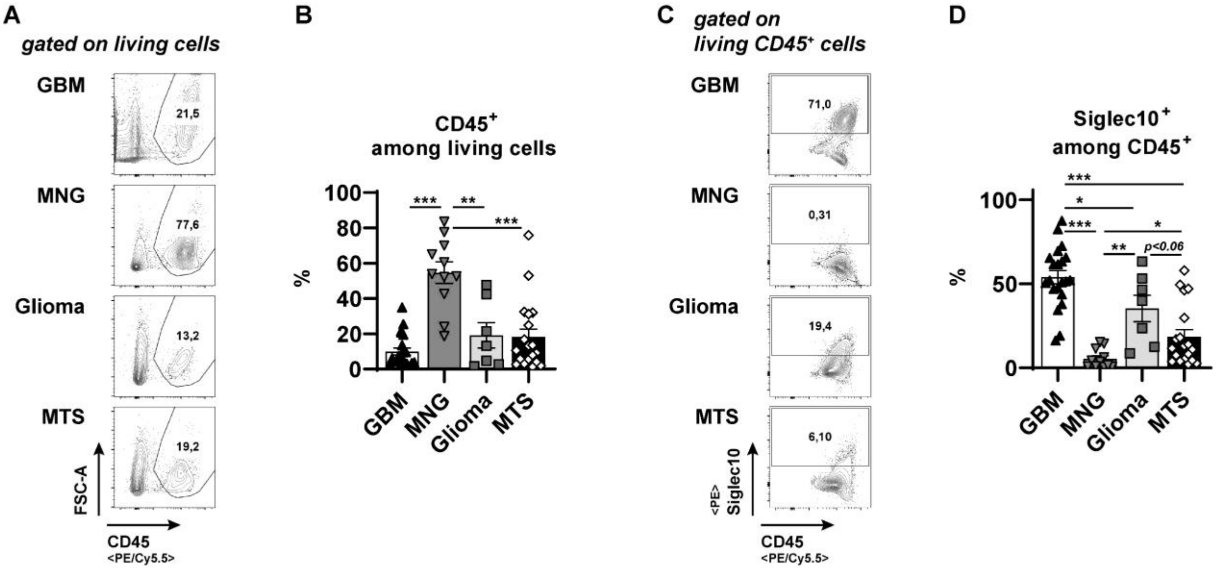

Suppl. Fig. 8: Enhanced Siglec10$^+$ cell frequencies among hematopoietic cells in GBM tumor environment. Tumor samples of the indicated entities for (GBM: n = 21; MNG: n = 12; Glioma: n = 7; MTS: n = 20) were digested and single cells were stained with antibodies against CD45 and Siglec10. **A)** Representative dot plot show gating on CD45$^+$ cells among living hematopoietic cells. **B)** CD45$^+$ cell frequencies were summarized among living cells of the indicated tumor entities. **C)** Representative plots for Siglec10 frequencies among living CD45$^+$ cells of the indicated tumor entities. **D)** Summarized Siglec10$^+$ frequencies among living CD45$^+$ cells of the indicated entities. Statistics: $^{*}p<0.05$, $^{**}p<0.01$, $^{***}p<0.001$

Supplement Figure 9

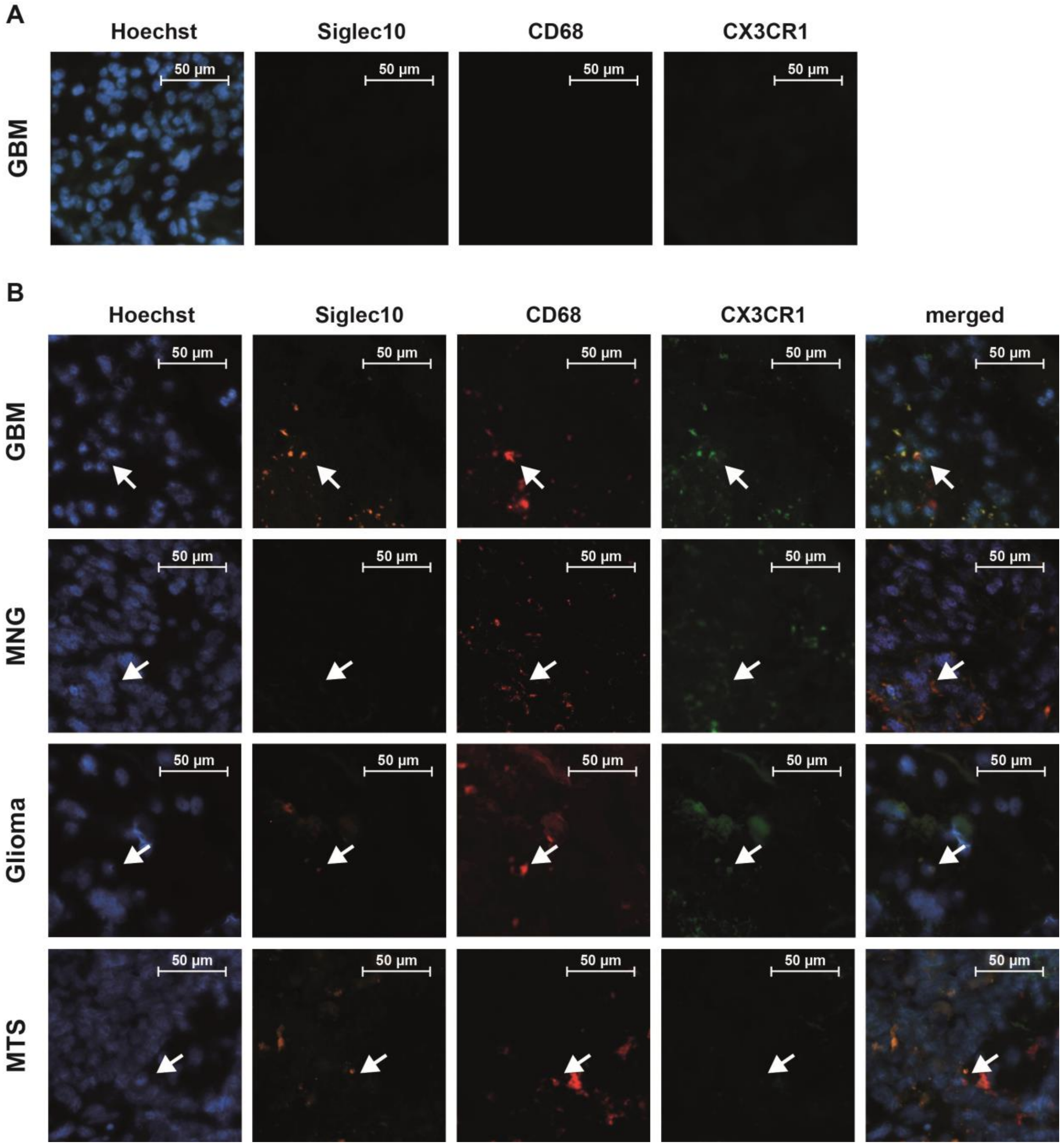

Suppl. Fig. 9: Enhanced Siglec10^{+} cell frequencies among hematopoietic cells in GBM tumor environment. Patient-derived tumor samples were stained with rabbit anti-human Siglec10, rat anti-human CX3CR1 and mouse anti-human CD68. Subsequently, fluorochrome-coupled antibodies were used for detections: AF488-conjugated anti-rat antibodies, AF568-conjugated anti-rabbit antibodies and Cy5-conjugated anti-mouse antibodies. Nuclei were stained with Hoechst33258. **A)** Negative controls were performed with the standard staining procedure by leaving out the unconjugated primary detection antibodies. Negative control stainings are shown for GBM histology sections. **B)** Representative pictures for each tumor entity are shown (n=5 independent patient samples per entity). The arrows indicate the locations of cells positive for CD68.

Supplement Figure 10

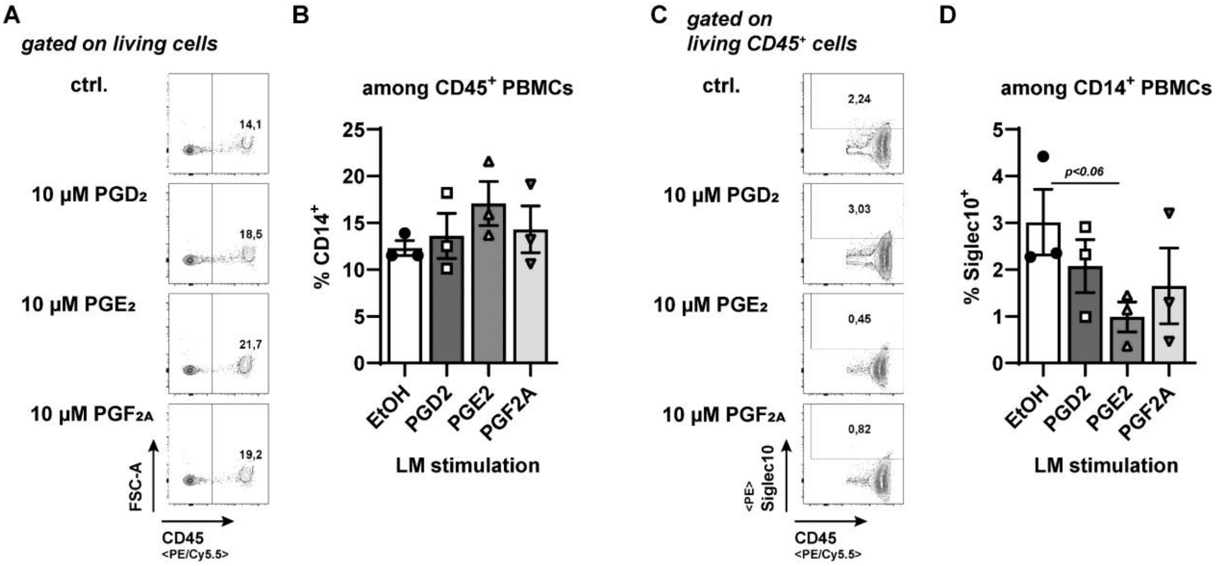

Suppl. Fig. 10: COX-induced prostaglandins do not induce Siglec10 expression on monocytes. PBMCs were isolated from healthy donors and cultured at $2x10^6$ cells/ well in a 24-well plate in 500 µL RPMI. Cells were stimulated with 10 µM PGD_2, PGE_2 or PGF_{2a} or with representative volume of EtOH (solvent control) for 24 h. Subsequently cells were recovered and analyzed for CD45, CD14 and Siglec10 expression by flowcytometry. Representative FACS plots are shown in (A) and (C). $CD14^+$ cells among living $CD45^+$ PBMCs are summarized in (B) and $Siglec10^+$ cells among living $CD14^+CD45^+$ monocytes are summarized in (D) (n=3 separate donors in 3 independent experiments).

Table 1 – Patient cohort

Patient #	sex	age at sampling	group	diagnosis	Analysis
1	female	71	GBM	glioblastoma	Tumor
2	male	65	non-GBM glioma	oligodendroglioma grade 3	Tumor
3	male	57	GBM	glioblastoma	Tumor
4	female	58	GBM	recurrent glioblastoma	Tumor
5	female	29	non-GBM glioma	recurrent astrocytoma grade 4	Tumor
6	male	84	MNG	meningioma grade 1, transitional subtype	Tumor
7	male	84	MNG	meningioma grade 1, transitional subtype	Tumor
8	male	61	MTS	NSCLC metastasis	Tumor
9	female	63	MTS	SCLC metastasis	Tumor
10	female	84	GBM	glioblastoma	Tumor
11	female	74	GBM	recurrent glioblastoma	Tumor
12	male	80	MTS	NSCLC metastasis	Tumor
13	male	73	MNG	meningioma grade 2	Tumor
14	female	53	GBM	glioblastoma	Tumor
15	female	10	non-GBM glioma	recurrent astrocytoma grade 1, pilocytic	Tumor
16	female	73	GBM	glioblastoma	Tumor
17	female	52	MTS	NSCLC metastasis	Tumor
18	male	59	MTS	melanoma metastasis	Tumor
19	female	71	MTS	melanoma metastasis	Tumor
20	female	87	GBM	glioblastoma	Tumor + Mono
21	female	87	MNG	meningioma grade 1	Tumor + Mono
22	male	65	MTS	MTS colon carcinoma	Tumor + Mono
23	female	75	MTS	NSCLC metastasis	Tumor + Mono
24	female	76	MTS	melanoma metastasis	Tumor + Mono
25	male	7	non-GBM glioma	astrocytoma grade 1, pilocytic	Tumor + Mono
26	male	69	GBM	glioblastoma	Tumor + Mono
27	male	40	non-GBM glioma	recurrent astrocytoma grade 3	Tumor + Mono
28	female	79	GBM	glioblastoma	Tumor + Mono
29	male	59	MTS	recurrent melanoma metastasis	Tumor + Mono
30	male	78	GBM	glioblastoma	Tumor + Mono
31	male	72	MNG	meningioma grade 1	Tumor + Mono
32	male	67	MTS	NSCLC metastasis	Tumor + Mono
33	male	48	MNG	meningioma grade 3, anaplastic	Tumor + Mono
34	male	69	MTS	kidney carcinoma metastasis	Tumor + Mono
35	female	40	MNG	meningioma grade 1	Tumor + Mono
36	female	77	MNG	meningioma grade 1	Tumor + Mono
37	male	64	MTS	melanoma metastasis	Tumor + Mono
38	male	64	MTS	melanoma metastasis	Tumor + Mono
39	male	61	MNG	meningioma grade 1	Tumor + Mono
40	male	65	GBM	recurrent glioblastoma	Tumor + Mono
41	female	55	non-GBM glioma	recurrent oligodendroglioma grade 3	Tumor + Mono
42	female	59	MNG	meningioma grade 1	Tumor + Mono
43	male	73	MTS	unknown metastasis	Tumor + Mono
44	male	66	non-GBM glioma	recurrent oligodendroglioma grade 3	Tumor + Mono
45	male	60	MTS	NSCLC metastasis	Tumor + Mono
46	female	60	GBM	glioblastoma	Tumor + Mono
47	female	57	MNG	meningioma grade 1	Tumor + Mono
48	male	37	non-GBM glioma	astrocytome grade 3	Tumor + Mono
49	male	55	GBM	recurrent glioblastoma	Tumor + Mono
50	female	65	MNG	meningioma grade 2	Tumor + Mono
51	male	88	MNG	recurrent meningioma grade 1	Tumor + Mono
52	male	68	GBM	glioblastoma	Tumor + Mono
53	female	60	GBM	glioblastoma	Tumor + Mono
54	male	42	MTS	NSCLC metastasis	Mono
55	female	65	MTS	NSCLC metastasis	Mono
56	male	42	MTS	NSCLC metastasis	Mono
57	male	80	GBM	glioblastoma	Tumor + Mono
58	male	72	GBM	recurrent glioblastoma	Mono
59	male	46	MTS	melanoma metastasis	Mono
60	male	46	MTS	melanoma metastasis	Mono
61	female	58	MNG	meningioma grade 1	Mono
62	male	65	GBM	recurrent glioblastoma	Mono
63	male	69	GBM	recurrent glioblastoma	Mono
64	male	65	GBM	recurrent glioblastoma	Mono
65	male	72	GBM	recurrent glioblastoma	Mono
66	male	52	GBM	glioblastoma	Mono
67	male	66	MTS	NSCLC metastasis	Mono
68	male	14	non-GBM glioma	glioneural tumor, grade 1	Mono
69	male	63	GBM	glioblastoma	Mono
70	female	65	MTS	Cervix Carcinoma metastasis	Mono
71	female	61	GBM	glioblastoma	Mono
72	female	84	GBM	glioblastoma	Mono
73	male	60	MTS	NSCLC metastasis	Mono
74	male	87	MTS	NSCLC metastasis	Mono
75	male	65	GBM	glioblastoma recidive	Mono
76	male	66	GBM	glioblastoma	Mono
77	male	42	non-GBM glioma	astrocytoma grade 4	Mono
78	male	62	MTS	liposarcoma metastasis	Mono
79	female	88	MNG	meningioma grade 1	Mono
80	female	47	non-GBM glioma	astrocytoma grade 3	Mono
